# Peripatric speciation within *Torreya fargesii* (Taxaceae) in the Hengduan Mountains inferred from multi-loci phylogeography

**DOI:** 10.1186/s12862-023-02183-1

**Published:** 2023-12-12

**Authors:** Yixuan Kou, Dengmei Fan, Shanmei Cheng, Yi Yang, Meixia Wang, Yujin Wang, Zhiyong Zhang

**Affiliations:** 1 Key Laboratory of Ecology of Rare and Endangered Species and Environmental Protection, Guangxi Normal University, Ministry of Education, Guilin, China; 2 Guangxi Key Laboratory of Landscape Resources Conservation and Sustainable Utilization in Lijiang River Basin, Guangxi Normal University, Guilin, China; 3 Laboratory of Subtropical Biodiversity, Jiangxi Agricultural University, Nanchang, China; 4 State Key Laboratory of Grassland Agro-Ecosystems, School of Life Sciences, Lanzhou University, Lanzhou, China

**Keywords:** Peripatric speciation, Population colonization, Ecological differentiation, The Hengduan Mountains, *Torreya fargesii*

## Abstract

**Background:**

The Hengduan Mountains (HDM) are one of the major global biodiversity hotspots in the world. Several evolutionary scenarios, especially *in-situ* diversification, have been proposed to account for the high species richness of temperate plants. However, peripatric speciation, an important mode of allopatric speciation, has seldom been reported in this region.

**Results:**

Here, two chloroplast DNA regions and 14 nuclear loci were sequenced for 112 individuals from 10 populations of *Torreya fargesii* var. *fargesii* and 63 individuals from 6 populations of *T. fargesii* var. *yunnanensis*. Population genetic analyses revealed that the two varieties are well differentiated genetically (*F*_ST_, 0.5765) and have uneven genetic diversity (*π*, 0.00221 vs. 0.00073 on an average of nuclear loci). The gene genealogical relationship showed that *T. fargesii* var. *yunnanensis* is inferred as derived from *T. fargesii* var. *fargesii*, which was further supported by the coalescent simulations (DIYABC, fastsimcoal2 and IMa2). By the coalescent simulations, the divergence time (~ 2.50–3.65 Ma) and the weak gene flow between the two varieties were detected. The gene flow was asymmetrical and only occurred in later stages of divergence, which is caused by second contact due to the population expansion (~ 0.61 Ma) in *T. fargesii* var. *fargesii*. In addition, niche modeling indicated that the two varieties are differentiated geographically and ecologically and have unbalanced distribution range.

**Conclusions:**

Overall, *T. fargesii* var. *fargesii* is always parapatric with respect to *T. fargesii* var. *yunnanensis*, and the latter derived from the former in peripatry of the HDM following a colonization from central China during the late Pliocene. Our findings demonstrate that peripatric speciation following dispersal events may be an important evolutionary scenario for the formation of biodiversity hotspot of the HDM.

**Supplementary Information:**

The online version contains supplementary material available at 10.1186/s12862-023-02183-1.

## Background

Allopatric speciation is widely recognized as the predominant mode of speciation [1, 2] in which divergence between populations is facilitated by geographic separation that prevents gene flow between them [2, 3]. Within the context of allopatric speciation, many authors advocate the importance of small founder populations and predict that new species tend to form as small range fragments around a widely distributed ancestral species (peripatric speciation) [4–6]. Since the endemic species on oceanic islands must have originated by colonization events from their closest relatives inhabiting a nearby continent, island species have provided exemplars for peripatric speciation [2]. Likewise, due to the linearly geological isolation on archipelagos, phylogeographic studies of endemic species on oceanic archipelagos have illustrated consistent patterns of sequential colonization and peripatric speciation along island chains [7]. Nevertheless, the occurrence of peripatric speciation on continents is formidable to ascertain since another form of allopatric speciation, vicariant speciation, is equally likely [2], and historical range connections might have experienced by the present-day isolated populations [6, 8]. By far, only a paucity of peripatric speciation events on continents have been documented in global biodiversity hotspots such as Amazonia [9], Eastern North America [10] and the Eastern Afromontane [11], while robust cases of peripatric speciation are generally lacking in the Hengduan Mountains (HDM), a region with exceptionally high species richness and known as a natural laboratory for the study of the origins, evolution and dispersal of temperate plant diversity [12].

Being located at the southeastern margin of the Qinghai-Tibetan Plateau (QTP), the HDM is one of the most unusual global biodiversity hotspots in the world. With only one fifth of the QTP’s area (ca. 500,000 km^2^), the HDM harbors a vascular flora of about 12,000 species, more than 3,300 being endemic [12–15]. The first and most prevailing evolutionary scenario accounting for the high species richness may be *in-situ* diversification (or vicariant speciation) associated with the recent and rapid tectonic uplift between the late Miocene and late Pliocene (ca. 10–2.6 Ma) [16–18]. Specifically, the tectonic uplift has shaped a highly heterogeneous geomorphology with the island-like isolation of numerous high peaks and ridges and produced a wide diversity of habitats, resulting in population divergence and allopatric speciation by physical isolation and local adaptation. The second scenario is the “flickering connectivity system” (FCS) that was recently proposed by Flantua and Hooghiemstra [19]. This scenario is focused on the connectivity dynamics during the glacial-interglacial cycles since the Quaternary (2.6 million before present), which may have fostered the adaptive divergence and speciation through repeated cycles of genetic admixture among populations followed by geographic isolation (a model also called Mixing-Isolation-Mixing, MIM) [20]. The third scenario is associated with gene flow between diverging populations, which may result in natural hybrids that could directly develop into new species through hybrid polyploidization and homoploid hybrid speciation (HHS) [18].

Although the plausibility of the above evolutionary scenarios, radiations within the HDM occurred predominantly in evolutionarily young taxa and/or species-rich genera, such as *Pedicularis*, *Gentiana*, *Saxifraga* and *Rhododendron* [18, 21]. Those genera always feature narrowly distributed species, sometimes endemic to a single mountain or valley [22]. For evolutionarily more stable and/or older lineages (such as many woody genera), however, the above evolutionary scenarios might be less likely within the HDM, because *in-situ* diversification for such taxa requires much larger area due to their large population sizes and extensive gene flow, and flickering connectivity with their relatives is irrelevant because those genera are often species-poor [23]. However, the strong uplift not only provides numerous opportunities for *in-situ* diversification and speciation through flickering connectivity within the HDM, but also offers a variety of free available niches for the colonization from adjacent floras (i.e., the Himalaya, QTP platform and Sino-Japanese floras), which may have a significant contribution to the biotic assembly of the HDM [12, 16, 17]. Given that the area of HDM is relatively small (50,000 km^2^) where is analogous to an island and where the rapidly uplift-induced alpine habitats may exert strong selection on newly colonized populations, and thus, the founder effect interacting with selection might initiate peripatric speciation [24]. To our best knowledge, explicit tests of the occurrence of peripatric speciation in the HDM have not been implemented, although a few cases of allopatric speciation which do not differentiate peripatric and vicariant speciation have been reported (e.g., [21, 25–27]). Such studies are critically needed to unravel the diversity of speciation mode within the HDM and to deepen our understanding of the origin and evolution of the world’s richest temperate flora [12, 17].

Although theoretically intuitive [2, 28], distinguishing different geographical modes of speciation is challenging. Traditionally, species-level phylogenies reconstruct the pattern of cladogenesis leading to extant species, and so the geographical ranges of sister clades identified from the phylogeny can be used to infer the geographical mode of speciation [6]. The key assumption underlying this approach is that the geographical range of both extant and ancestral species at the time of speciation can be inferred from present-day distributions [6, 8]. However, one disadvantage of this approach is that the current distribution of species is not a reliable indicator of the historical geographical range of the same species because changes in distribution may happen over short periods of time due to climate changes, colonization of new areas, extinction of competitors, among others [8, 29]. In addition, phylogenetic approaches can not incorporate demographic parameters (e.g., effective population size, divergence time, direction and magnitude of gene flow) to discriminate between different geographical models of speciation within allopatric speciation. When geographical isolation drives speciation (allopatric speciation), a complete termination of gene flow for a prolonged period will occur immediately after the formation of the geographical barrier between diverging populations. If speciation is driven by ecologically divergent selection in sympatry or parapatry (sympatric speciation or parapatric speciation), gene flow of selectively neutral genomic regions may go on between diverging populations until the completion of reproductive isolation [2, 28]. When considering peripatric speciation specifically, one should expect to find smaller effective population sizes of species in peripatry than those of their sister species, a lack of gene flow after speciation, and significant asymmetry in range size between sister species [30]. Coalescent approaches in population genetics have been developed since the past 40 years to examine the historical processes responsible for patterns of genetic variation that exist within and among populations (reviewed in [31]). Several coalescent methods, such as Approximate Bayesian Computation (ABC, [32]), Isolation with Migration (IM, [33]), and faster continuous-time sequential Markovian coalescent algorithm (fastsimcoal2, [34]) which have been developed primarily to test demographic, genetic and ecological mechanisms of speciation, have provided deep insights into the process of speciation, particularly coalescent analyses of multiple independent loci or genomic data with standard phylogeographic analyses [35, 36].

**Fig. 1:**
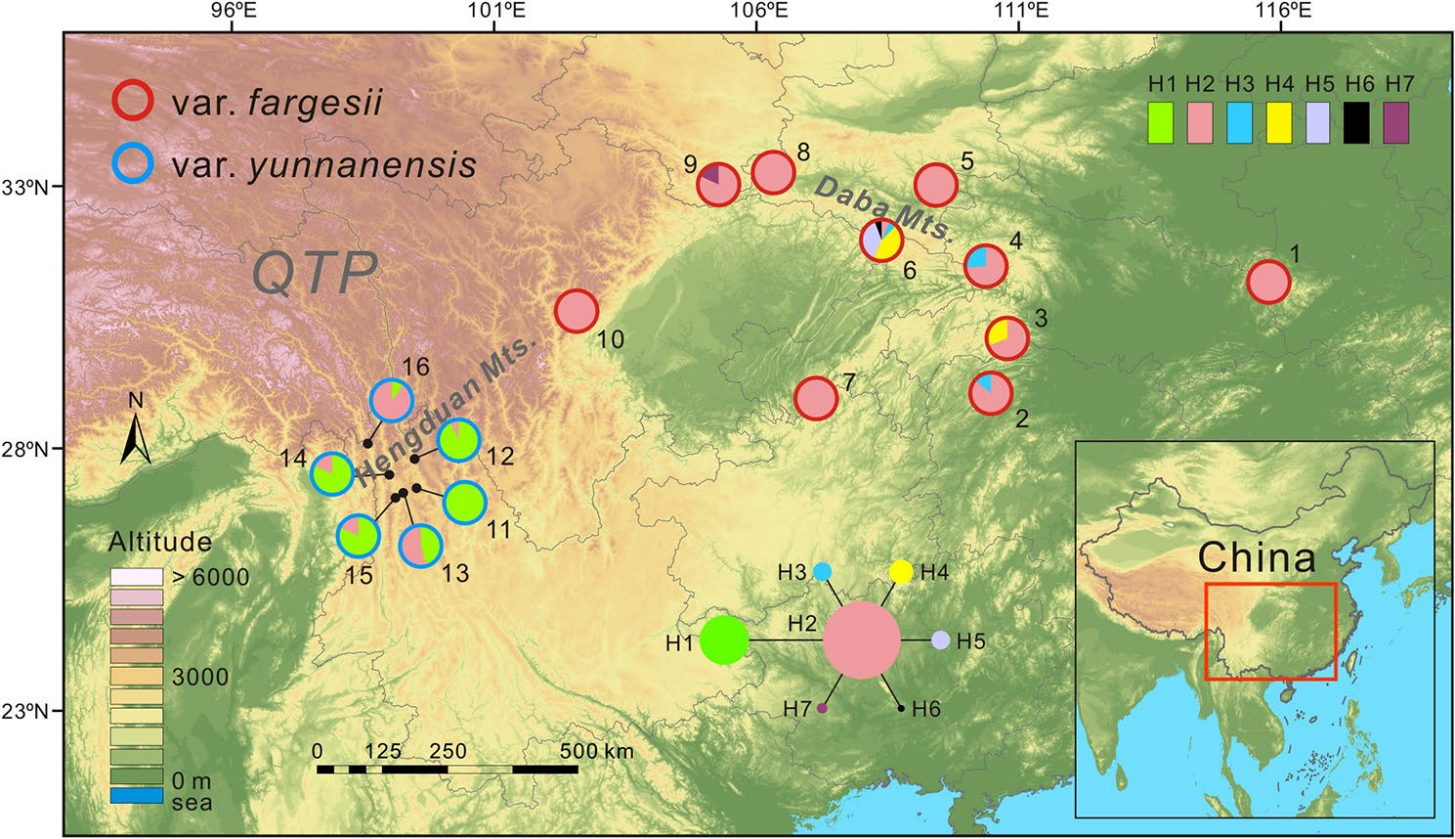
Geographical distribution and network of seven chloroplast haplotypes within *T. fargesii* var. *fargesii* and *T. fargesii* var. *yunnanensis*. The sizes of circles in the network are proportional to the haplotype frequencies, and one mutation step is among each derived haplotype and the central one. QTP, Qinghai–Tibetan Plateau. The base map (altitude layer) was downloaded from WorldClim database (https://www.worldclim.org) and the colored pies were drawn in ArcGIS v10.0 (ESRI, Redlands, CA, USA)

*Torreya fargesii* Franch. (Taxaceae) is an endemic and endangered conifer species in China [37–39] belonging to an ancient and species-poor genus *Torreya* Arn. (divergence age from *Amentotaxus* inferred around 54.74 million years ago and containing six species distributed in eastern Asia and North America) [40]. As common in conifers, *T. fargesii* is wind-pollinated and has a quite large distribution range in central China (Fig. 1). Two varieties (*T. fargesii* var. *fargesii* and *T. fargesii* var. *yunnanensis*) within the species are currently recognized according to the differences in leaf traits (length: 1.5–3 cm vs. 2–3.6 cm; leaf form: straight vs. strongly falcate) and seed morphology (no longitudinal ridges on the inner wall of testa vs. two longitudinal ridges) [37, 41]. However, it should be noted that *T. fargesii* var. *yunnanensis* was originally published as an independent species (*Torreya yunnanensis* Cheng et L.K. Fu, [42]). *T. fargesii* var. *fargesii* extensively distributes in the surrounding mountains of the Sichuan Basin in central China, particularly abundant in Daba Mountains, a core region of the Sino-Japanese Flora which contain many ancient and relict conifer species such as *Metasequoia glyptostroboides* and *Ginkgo biloba* [43]. On the contrary, *T. fargesii* var. *yunnanensis* is restricted to the HDM (Fig. 1) [44, 45], which belongs to the much younger Sino-Himalayan Flora [46], a center of temperate plant diversification in the world [47]. A few phylogenetic analyses indicated that the two varieties might be recently diverged lineages representing two incipient species [48–51]. During the past two decades, the field of speciation genetics has been progressed from model systems of hybrid inviability between relatively distant species to natural systems undergoing incipient speciation [52]. Thus, the two varieties of *T. fargesii* might stand for an ideal system for speciation genetic studies in *Torreya.* However, the divergence and speciation history of the two varieties have not been explored by means of coalescent-based population genetics. Given the asymmetrical distribution ranges of the two varieties and the relatively young geological history of the HDM, we postulate that *T. fargesii* var. *yunnanensis* might have resulted from a peripatric speciation event that happened in the HDM, following a colonization from the central China. To test this hypothesis, we combined three coalescent-based population genetic approaches (ABC, IM, and fastsimcoal2) and niche modelling to elucidate the speciation history of *T. fargesii* var. *yunnanensis* and *T. fargesii* var. *fargesii.* The results of this study will shed new insights into the formation of species diversity in the HDM.

## Results

### Genetic diversity and neutrality tests

Seven cpDNA haplotypes were inferred from one indel and five substitutions in two chloroplast regions, *trn*L-*trn*F (901 bp) and *rpo*B-*trn*C (688 bp) (Additional file 1: Table S1). The within-population haplotype diversity (*H*_d_) and nucleotide diversity (*π*) ranged from 0 to 0.7 and 0 to 0.00072 in *T. fargesii* var. *fargesii*, and from 0 to 0.5455 and 0 to 0.00034 in *T. fargesii* var. *yunnanensis*, respectively. The total *H*_d_ and *π* of *T. fargesii* var. *yunnanensis* (0.4403 and 0.00028) were slightly higher than those of *T. fargesii* var. *fargesii* (0.3982 and 0.00026) (Additional file 1: Table S2).

Fourteen low-copy nuclear loci were sequenced and aligned across all samples of *T. fargesii* with a total length of 6,077 bp. Six indels, three in *T147* and three in *T203*, were detected and excluded in subsequent analyses. The level of genetic diversity showed significant difference between the two varieties (Additional file 1: Table S3). A total of 174 segregating sites (*S*) were detected in 14 nuclear loci in *T. fargesii* var. *fargesii*, but 30 in *T. fargesii* var. *yunnanensis*; Numbers of haplotype (*H*_h_) within populations varied from 3 to 38 (mean = 13.64) in the former, but from 1 to 7 (mean = 2.86) in the later. The average haplotype diversity (*H*_d_), nucleotide diversity (*π*) and Watterson’s parameter (*θ*_w_) across 14 nuclear loci were 0.476, 0.00221 and 0.0045 in *T. fargesii* var. *fargesii*, markedly higher than those in *T. fargesii* var. *yunnanensis* (0.233, 0.0007 and 0.0009, respectively). A similar pattern was also observed in the nonsynonymous sites and silent sites. Seven minimum number of recombinant events (*R*_m_) were detected in 14 nuclear loci in *T. fargesii* var. *fargesii*, but only one in *T. fargesii* var. *yunnanensis*.

The majority of nuclear loci showed negative values of Tajima’s *D* and Fu and Li’s D* and F* in both varieties, with five (*T8*, *T147*, *T173*, *T203*, *T222* and *T235*) being significantly deviated from neutral expectation (*P* < 0.05). In Fay and Wu’s *H* test, *T8* and *T203* deviated from the neutral model (*P* < 0.05). The mean values of Fay and Wu’s *H* were negative for *T. fargesii* var. *yunnanensis*, but positive for *T. fargesii* var. *fargesii*. MFDM tests failed to detect the likelihood of natural selection acting on individual loci, with the exception of two loci *T8* and *T147* (*P* < 0.05) in *T. fargesii* var. *fargesii* (Additional file 1: Table S4). However, the multilocus Hudson-Kreitman-Aguadé (HKA) tests showed that no locus deviated from neutrality in each variety (*T. fargesii* var. *fargesii*, *χ*^2^ = 13.8855, *P* = 0.38196; *T. fargesii* var. *yunnanensis*, *χ*^2^ = 16.1728, *P* = 0.23992). Therefore, two datasets, one included all nuclear loci and the other excluded loci *T8* and *T147*, were assembled and used separately for inferring demographic history of *T. fargesii*.

### Haplotype network and distribution

The median-joining network of chloroplast DNA displays a star-like pattern. H2 was in the central position and other haplotypes connected to H2 each by one mutation (Fig. 1). H2 was shared by the two varieties, one haplotype (H1) was private to *T. fargesii* var. *yunnanensis* and five (H3–H7) to *T. fargesii* var. *fargesii* (Fig. 1, Additional file 1: Table S2). All populations of *T. fargesii* var. y*unnanensis* contained two haplotypes (H1 and H2) except for population 11 (only H1). In contrast, H2 predominated the populations of *T. fargesii* var. *fargesii*, with other minor haplotypes being private or shared by two or three populations). Notably, population 6 in the Daba Mountains had an exceptionally high number of haplotypes (H2, H3, H4, H5 and H6) (Fig. 1, Additional file 1: Table S2).

**Fig. 2:**
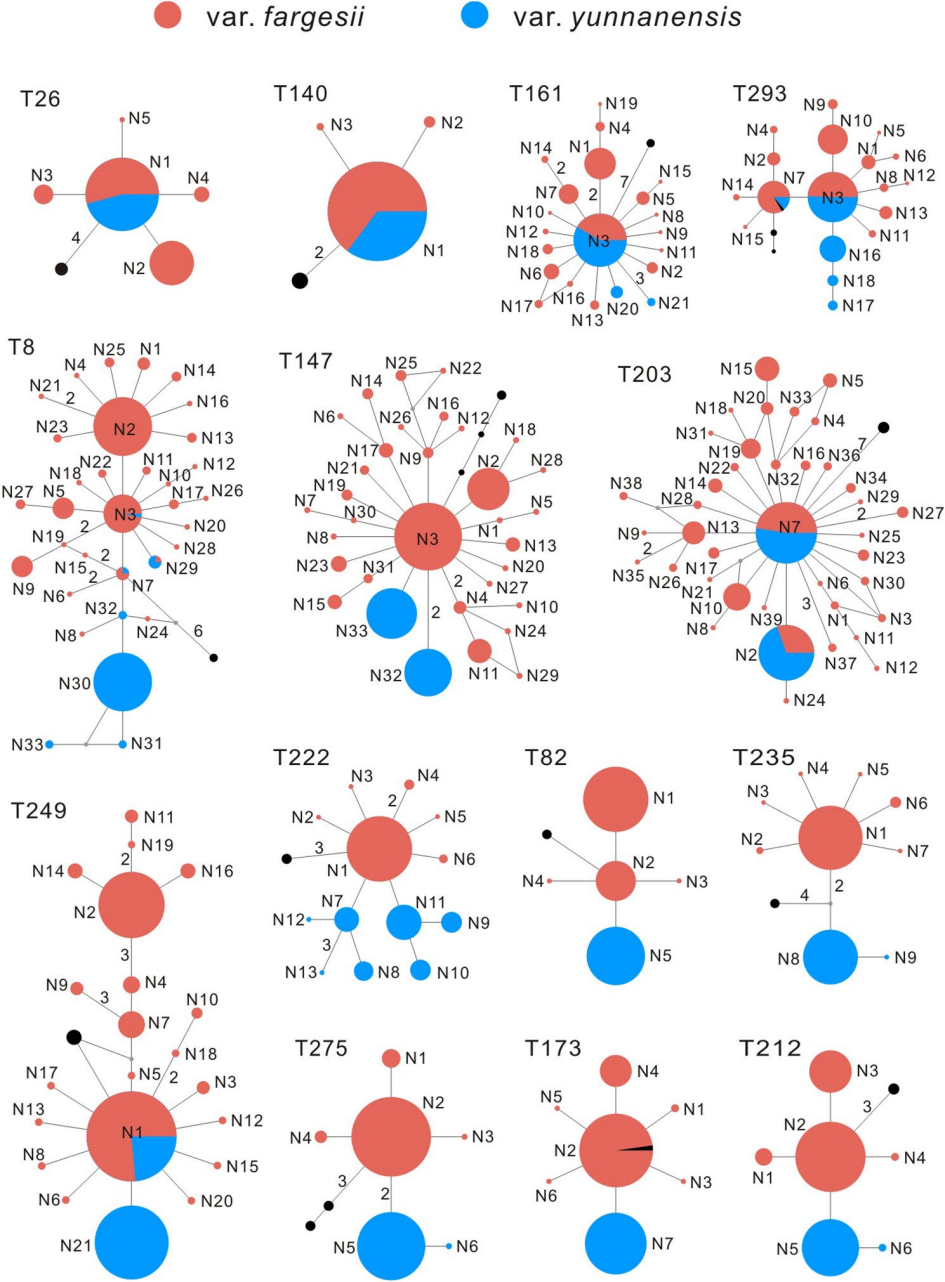
Haplotype genealogies of the fourteen nuclear loci for *T. fargesii* var. *fargesii* and *T. fargesii* var. *yunnanensis*. The sizes of circles are proportional to the haplotype frequencies, and the mutation steps more than one are marked the corresponding number on each branch. The haplotypes (black circles) of *T. taxifolia* are used as the outgroup

Overall, *T. fargesii* var. y*unnanensis* harbored much less haplotypes than *T. fargesii* var. *fargesii* did for each 14 nuclear loci. There were three types of nuclear haplotype networks (Fig. 2): (1) each variety had well differentiated and private haplotypes (Locus *T235* and *T275*); (2) *T. fargesii* var. y*unnanensis* had private haplotypes, which were derived from *T. fargesii* var. *fargesii* by one mutation (*T82*, *T147*, *T173*, *T212* and *T222*); (3) *T. fargesii* var. *yunnanensis* had less haplotypes (except for *T222*) and one to three haplotypes were shared between the two varieties (*T8*, *T26*, *T140*, *T161*, *T203*, *T249* and *T293*).

### Population genetic structure

**Fig. 3:**
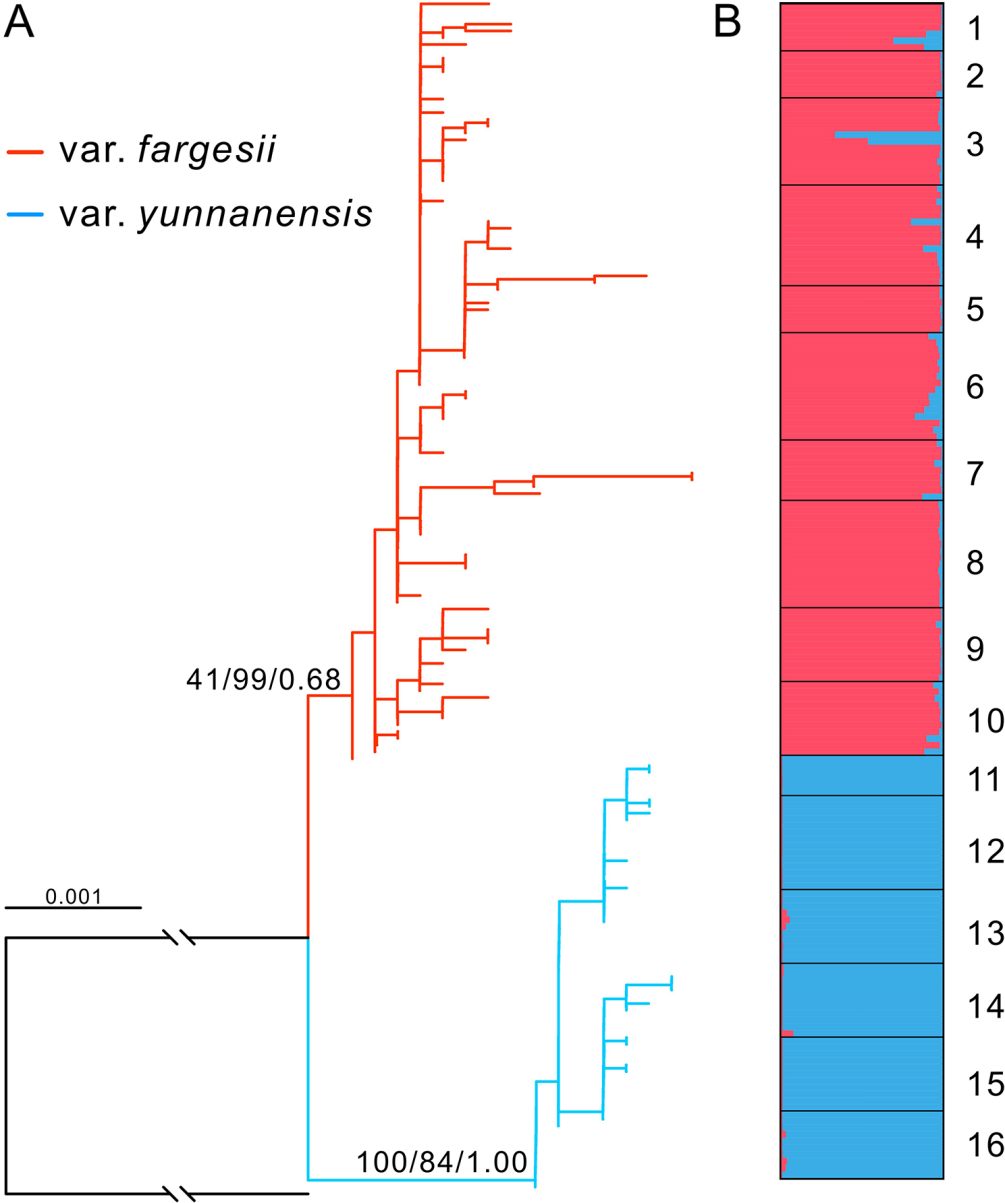
The relationship between *T. fargesii* var. *fargesii* and *T. fargesii* var. *yunnanensis* was inferred using ML phylogenetic tree (**A**) and Structure analysis (**B**). The numbers above the branches indicate the support values of ML, NJ, and Bayesian trees. The numbers 1 to 16 on the right of Structure histogram correspond separately to the population codes in Fig. 1

The genetic relationships among individuals of the two varieties were explored by constructing phylogenetic trees based on the concatenated nuclear loci. The ML tree revealed that all individuals were divided into two clades corresponding exactly to the two varieties (Fig. 3A). The same topology between the two varieties was also inferred in Neighbor–Joining (NJ) and Bayesian trees (Additional file 1: Fig. S1). The clade of *T. fargesii* var. *yunnanensis* received high support values (100/99/1.00), but *T. fargesii* var. *fargesii* were moderately supported (41/99/0.68) (Fig. 3A). However, a polyphyletic clade of *T. fargesii* var. *fargesii* and a monophyletic clade of *T. fargesii* var. *yunnanensis* were revealed in the phylogenetic tree estimated using the partitioned nuclear data, and the latter is nested within the former (Additional file 1: Fig. S2). This result indicated that *T. fargesii* var. *yunnanensis* was probably derived from *T. fargesii* var. *fargesii*, but the posterior values for the clades in the tree are very low (< 0.5).

Consistent with the phylogenetic analyses, two distinct clusters (*K* = 2) were revealed using the Bayesian clustering algorithm based on 152 independently segregate sites. *T. fargesii* var. *fargesii* had ~ 4% genetic component of *T. fargesii* var. *yunnanensis*, while ~ 1% *T. fargesii* var. *fargesii* component in *T. fargesii* var. *yunnanensis* (Fig. 3B, Additional file 1: Fig. S3). The genetic differentiation (*F*_ST_) between the two varieties for each nuclear locus showed significant difference ranging from 0.1156 (*T203*) to 0.9777 (*T275*) (*P* < 0.01) with exception of locus *T140* (-0.00352, *P* > 0.05), the *F*_ST_ across all loci was also significant (0.5765, *P* < 0.01) (Additional file 1: Table S5).

### Divergence and demographic history

According to the results of neutrality test for each nuclear locus, two datasets were separately constructed to infer population divergence and demographic history (including effective population size, divergence time, migration rate, and change in population size) using three coalescent approaches, DIYABC, fastsimcoal2, and IMa2. One dataset (dataset I) included all nuclear loci, and the other (II) excluded loci *T8* and *T147* that are presumably under selection. However, the modeling results based on the two datasets were almost identical in the three coalescent approaches (Additional file 1: Tables S6–S15, Fig. S4), except that no migration was detected in fastsimcoal2 based on dataset II. In order to be consistent with the results from all analyses, only the results from dataset I were showed as follows.

**Fig. 4:**
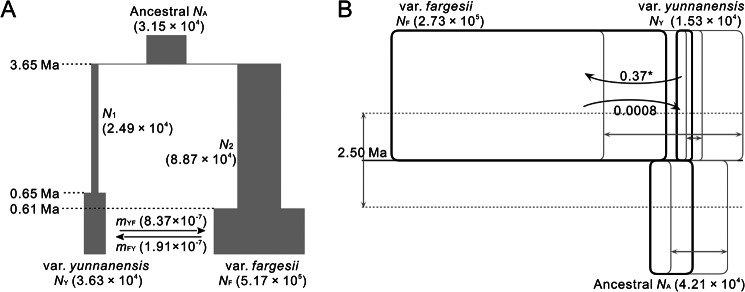
Demographical history of *T. fargesii* var. *fargesii* and *T. fargesii* var. *yunnanensis* was simulated by (**A**) coalescent simulation (fastsimcoal2) and (**B**) isolation-with-migration (IM) model. Effective population size (*N*_e_) for current and historical populations were depicted by the ratio of the width of the gray bars (**A**) or blank boxes (**B**), and the effective population sizes were showed in parentheses next to each population name; divergence time (Ma, million years ago) between the two varieties was presented on a horizontal dotted lines (**A**) or line (**B**); migration rate (*m* or 2*Nm*) were showed on a solid arrow, and the arrow indicated the direction of migration. The 95% highest posterior density intervals were showed with the double-sided arrows and blank boxes in grey for effective population size and divergence time in IM model (B). The detailed posterior interval for each demographic parameter can be found in Additional file 1: Table S11, S14. Asterisk (*) presented the migration rate at significant level (*P* < 0.05)

DIYABC modeling based on dataset I revealed that the B1 scenario (Additional file 1: Fig. S5) had the highest posterior probability (52.08% estimated by direct approach and 55.11% by logistic approach) and better reliability relative to other three basic scenarios (A1, B1, C1 and D1) (Additional file 1: Table S6, Figs. S6, S7). The simulation using fastsimicoal2 further supported that the B3 scenario (a derivative of B1 scenario, Additional file 1: Fig. S5) was the best one by comparing likelihood and AIC values (Additional file 1: Table S10), indicating that *T. fargesii* var. *yunnanensis* derived from a colonizing population of *T. fargesii* var. *fargesii* with recent migration due to a recent expansion of *T. fargesii* var. *fargesii* (Fig. 4A). Posterior estimates for demographic parameters (except for gene flow) were similar between DIYABC and fastsimcoal2 (Additional file 1: Tables S7, S11), so the statistics for demographic parameters from fastsimicoal2 is only showed as follows. The two varieties diverged around 3.65 million years ago (Ma, 95% CI: 3.51–3.80 Ma). The effective population size of *T. fargesii* var. *fargesii* (5.17 × 10^5^, 95% CI: 4.99–5.35 × 10^5^) was much larger than that of *T. fargesii* var. *yunnanensis* (3.63 × 10^4^, 95% CI: 3.38–3.87 × 10^4^). Population expansions were detected in both varieties, but stronger and more recent (0.61 Ma, 95% CI: 0.58–0.64 Ma) in *T. fargesii* var. f*argesii* and weaker and more ancient (0.65 Ma, 95% CI: 0.56–0.75 Ma) in *T. fargesii* var. *yunnanensis*. A similar pattern for their population expansions were also revealed in the analyses of Bayesian skyline plots (Additional file 1: Fig. S8). Gene flows (*m*) between the two varieties were weak, but from *T. fargesii* var. *yunnanensis* to *T. fargesii* var. *fargesii* (8.37 × 10^− 7^, 95% CI: 7.19–9.56 × 10^− 7^; 2*Nm* = 0.87) were stronger than vice versa (1.91 × 10^− 7^, 95% CI: 1.07–2.75 × 10^− 7^; 2*Nm* = 0.01) (Fig. 4A, Additional file 1: Table S11).

Demographic parameters estimated from IM model were overall lower than but still comparable with those from DIYABC and fastsimcoal2 (Fig. 4B, Additional file 1: Table S14). The divergence time between the two varieties was around 2.5 Ma (95% HPD: 1.58–3.39) based on dataset I. The effective population size of *T. fargesii* var. *fargesii* (2.73 × 10^5^, 95% HPD: 2.11–3.49 × 10^5^) was substantially larger than that of *T. fargesii* var. *yunnanensis* (1.53 × 10^4^, 95% HPD: 0.95–2.52 × 10^4^). Gene flow (2*Nm*) from *T. fargesii* var. *yunnanensis* to *T. fargesii* var. *fargesii* (0.3654, 95% HPD: 0.0030–1.9239) was much stronger than that of the reverse direction (0.0008, 95% HPD: 0–0.0109).

### Projected distribution with niche modeling and ecological differentiation

**Fig. 5:**
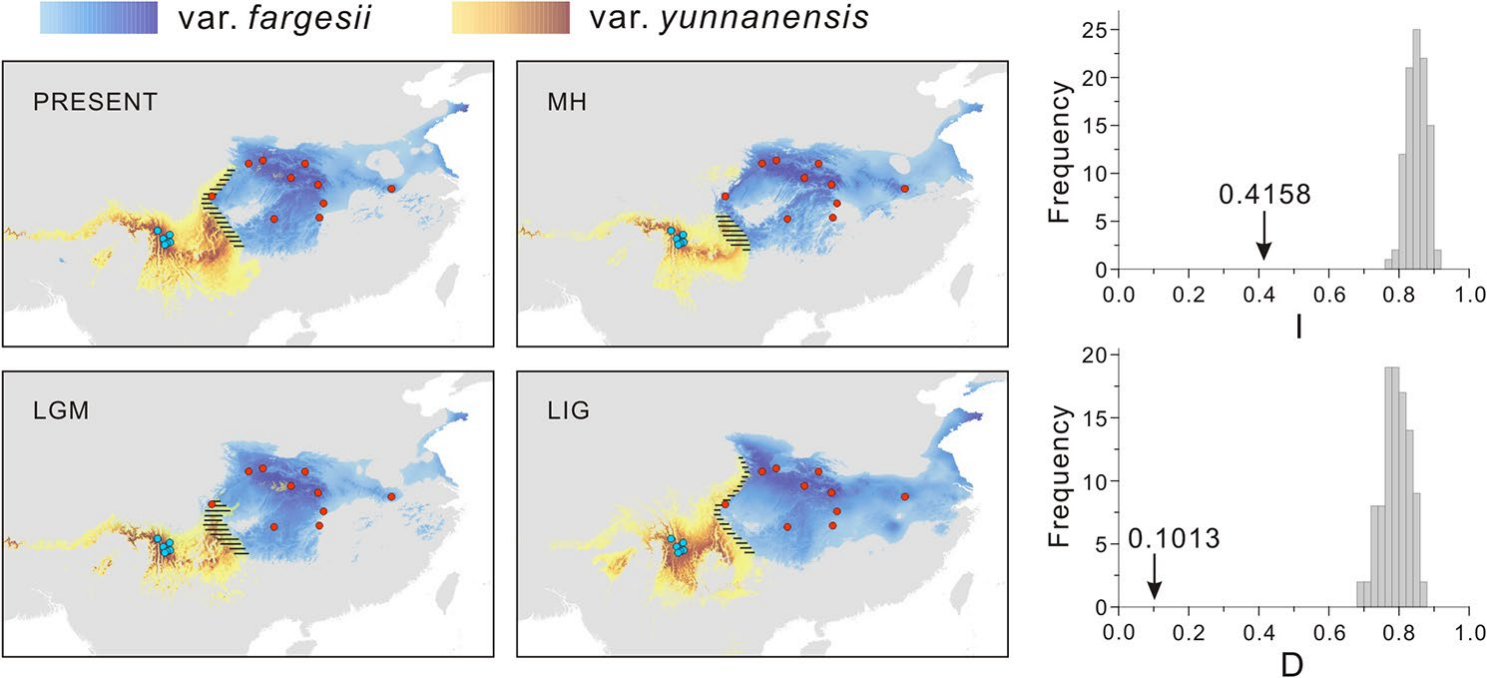
Climate niches modeled and drawn using MAXENT 3.4.3 and the niche difference measured by identity tests (I and D) for *T. fargesii* var. *fargesii* and *T. fargesii* var. *yunnanensis*. Predicted distributions are showed during present-day (PRESENT), Mid-Holocene (MH), the Last Glacial Maximum (LGM), and the Last Interglacial (LIG) climatic periods. Paleoclimate date for the MH, LGM and LIG are under MIROC model. Grey bars in identity tests indicate the null distributions of I and D, and arrow represents the actual values in Maxent runs. The overlaps between the predicted distributions of the two varieties were filled with solid lines. Red dots and blue dots represent the actual sampling records of *T. fargesii* var. *fargesii* and *T. fargesii* var. *yunnanensis*, respectively

**Fig. 6:**
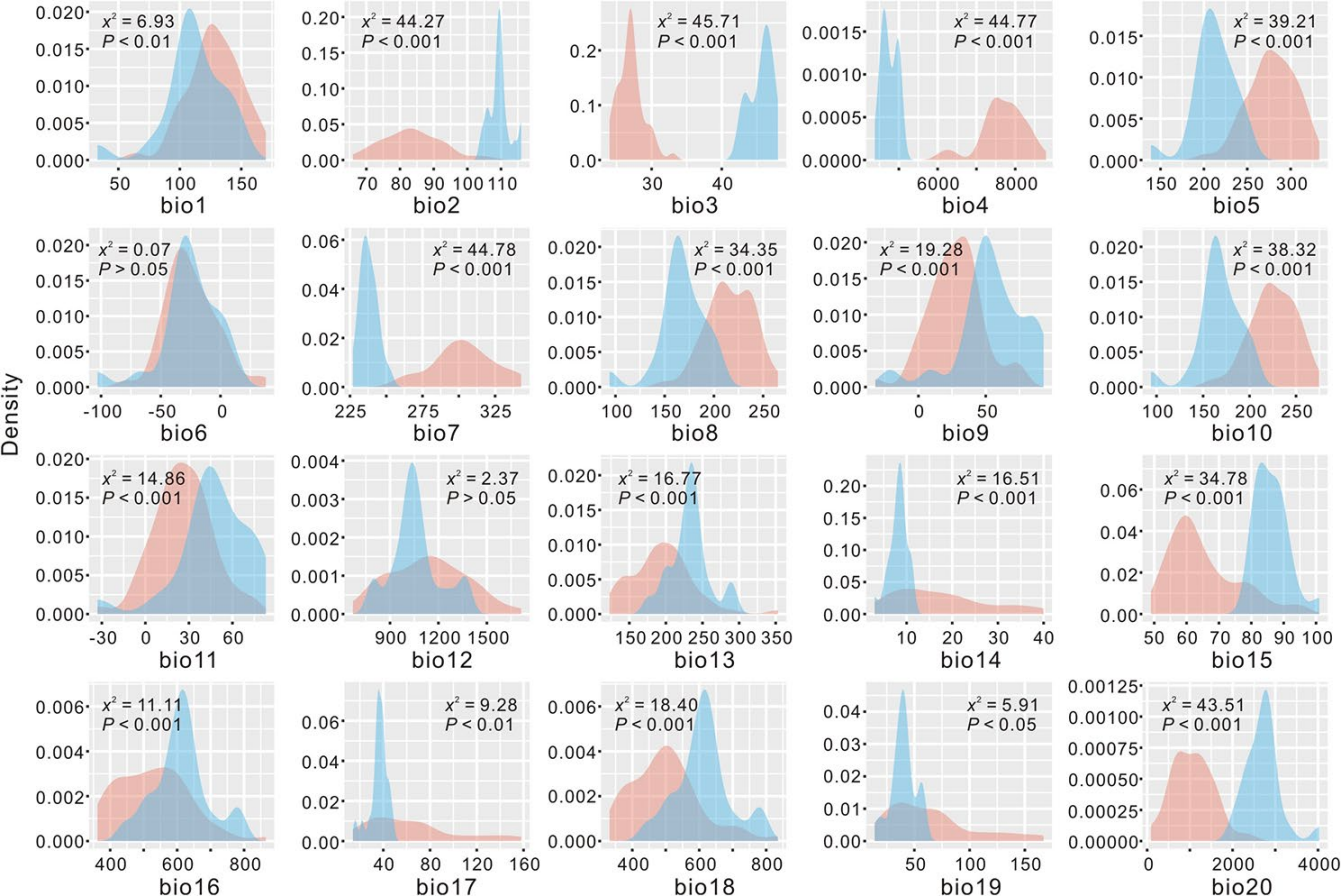
Kernel density plots for 19 climatic variables and altitude (bio20) of *T. fargesii* var. *fargesii* (red curves) and *T. fargesii* var. *yunnanensis* (blue curves). The differences of each ecological variable between the two varieties are assessed using nonparametric Kruskal-Wallis test and showed in each plot with *χ*^2^ and *P* value

MAXENT model predicted that *T. fargesii* var. *fargesii* had a wider present distribution than *T. fargesii* var. y*unnanensis*, and the niche models fitted well to the presence data with high AUC values (> 0.98) (Fig. 5, Additional file 1: Fig. S9). Moreover, except for the east of HDM where *T. fargesii* var. *yunnanensis* is absent at present, the predicted distributions accurately represented the extant distribution of the two varieties (Fig. 5). In addition, the projected distributions of the two varieties have changed very little since the LIG (Fig. 5, Additional file 1: Fig. S10), indicating that their distributions were relatively stable and weakly influenced by climate changes during the late Pleistocene. The results of ENMTOOLS showed that the observed values for I and D were significantly lower than those expected from pseudoreplicated data sets (*P* < 0.01) (Fig. 5), indicating the two varieties were well differentiated ecologically. The Kruskal-Wallis test displayed that almost all ecological variables were significantly different between the two varieties (*P* < 0.05), with exception of Mean Temperature of Coldest Month (bio6) and Annual Precipitation (bio12) (*P* > 0.05) (Fig. 6), enhancing the role of ecology in the divergence of the two varieties.

## Discussion

### *Torreya fargesii* var. *Fargesii* and *T. fargesii* var. *Yunnanensis* are two recently diverged species

Recently diverged species provide ideal materials for untangling the process of speciation [53, 54]. Previous phylogenetic studies on *Torreya* revealed that *T. fargesii* var. *fargesii* and *T. fargesii* var. *yunnanensis* are sister groups that received high supports [48–51], except for the study of Zhang et al. [40] where misidentification of *T. nucifera* already reported by Zhou et al. [51] could be responsible for their inference of the two varieties clustered within *T. nucifera*. In this study, we found that the two varieties of *T. fargesii* are well differentiated (Fig. 3) and should be treated as two independent species for several reasons.

First, the genetic differentiation (*F*_ST_) between the two varieties across 14 nuclear loci (0.5765, *P* < 0.01) was comparable with the values between other sister or close-related conifer species measured using multiple nuclear loci, such as, *Picea schrenkiana* and *P. smithiana* (0.6312, [55]), *Pinus massoniana* and *P. hwangshanensis* (0.4080, [56]), four *Picea* species (0.069 to 0.529, [57]), and four *Juniperus* species (0.2259 to 0.5304, [58]). Second, the divergence time between the two varieties was dated to the late Pliocene or early Pleistocene (2.50 Ma in IMa, 3.20 Ma in DIYABC, and 3.65 Ma in fastsimcoal2), these estimates are slightly younger than other incipient speciation events reported in *Taxus wallichiana* (4.2 Ma, [25]) and *Pinus armandii* (4.5 Ma, [59]) in the Himalaya-Hengduan Mountains. Third, although the two varieties share some ancestral haplotypes (at the center of the network) which are likely a result of incomplete lineage sorting (Figs. 1 and 2), there are many haplotypes fixed in the two varieties, for example, the chlorotype H1 being exclusive to *T. fargesii* var. *yunnanensis*, and some nuclear haplotypes (e.g., in genes *T235* and *T275*) being private to either of the two varieties. Fourth, there are ecologically significant differences found between the two varieties (Fig. 5), such as temperature (bio2, bio3, bio4, and bio7) and precipitation (bio15) (Fig. 6). These climatic differences could maintain the separation of the two varieties to some extent. Altogether, the divergence of the two varieties of *T. fargesii* could occur recently, and it is better to recognize the two varieties as two species in line with the taxonomic treatment of Cheng et al. [42].

### Peripatric speciation by population colonization in the Hengduan Mountains

Two models of allopatric speciation, peripatric speciation and vicariant speciation, are supposed to have a period of geographic isolation initially, which can be used to distinguish them from parapatric speciation [2]. But it is difficult to deduce whether the two varieties were isolated geographically at the initial stage of speciation from their present distributions, because the changes in geographical distribution could have been caused by a host of reasons (such as climate changes, colonization of new areas and extinction of competitors) [8, 29]. However, two lines of evidence could be used to support that the two varieties are the products of allopatric speciation. First, the inference of divergence history using fastsimcoal2 suggested that the two varieties could have went through a long period of strict allopatry initially and weak and asymmetrical gene flow only detected at the late stage of divergence, possibly caused by a recent demographic expansion in *T. fargesii* var. *fargesii* (Fig. 4A, Additional file 1: Tables S10, S12). Asymmetrical gene flow is generally considered to occur almost exclusively from the local to the invading species [60], and environment-specific fitness differences lead the introgressed genes to be eliminated in the local species through selection effects [61–64]. *T. fargesii* var. *yunnanensis* is limited in an extremely narrow area in the HDM (Fig. 5), indicating the variety highly exclusive to local environment relative to *T. fargesii* var. *fargesii*. Hence the fitness of introgressed genes from *T. fargesii* var. *fargesii* due to its population expansion into the territory occupied by *T. fargesii* var. *yunnanensis* may be reduced, resulting that the signals of gene flow from the former to the later were weaker than vice versa (Fig. 4A). Second, the projected distributions with niche modelling indicated that the core distributions of the two varieties were stable and had never overlapped with each other across the late Quaternary (Fig. 5). Although the results cannot be extrapolated to the late Pliocene when the two varieties began to diverge, it is highly likely that the two lineages cannot grow together due to their different climatic envelopes (also see discussion below).

Unlike vicariant speciation, peripatric speciation is often generated by colonizing populations with small sizes, and the colonizing populations always carry restricted genetic variation derived from the ancestral populations [1, 2, 65]. In line with the expectations of the peripatric speciation model, the study system herein presented the following features: (*i*) the distribution of *T. fargesii* var. *yunnanensis* is restricted to the HDM, much narrower than the distribution of *T. fargesii* var. *fargesii* (Figs. 1 and 5); (*ii*) much lower nuclear genetic diversity (*π*) and smaller effective population size (*N*_e_) were detected in the former (0.00073 and 3.63 × 10^4^) than in the latter (0.00221 and 5.17 × 10^5^) (Fig. 4, Additional file 1: Table S3). Note that total *H*_d_ and *π* of chloroplast DNA in *T. fargesii* var. *yunnanensis* (0.4403 and 0.00028) are slightly higher than those in *T. fargesii* var. *fargesii* (0.3982 and 0.00026), but the latter has much more haplotypes than the former (6 vs. 2) (Fig. 1, Additional file 1: Table S2); (*iii*) the gene genealogies of chloroplast and nuclear loci (except for *T235* and *T275*) showed that *T. fargesii* var. *yunnanensis* is inferred as derived from *T. fargesii* var. *fargesii* (Figs. 1 and 2). From the phylogenetic perspective, *T. fargesii* var. *fargesii* is inferred as sister clade with *T. fargesii* var. *yunnanensis* (Fig. 3, Additional file 1: Fig. S1) [48–51], a signature that is often resulted from the mode of peripatric speciation [2]; (*iv*) an ancestral-derivative scenario was supported by the coalescent simulations (Fig. 4, Additional file 1: Figs. S6, S7, Tables S10, S12). A similar pattern has also been observed in a well-known species pair on the continent of North America: red spruce (*Picea rubens*) and black spruce (*Picea mariana*). Due to the Pleistocene glaciations, a population of black spruce became geographically isolated from the major range of black spruce and is recognized as a distinct species (red spruce). The red spruce has significantly lower genetic diversity of both nuclear DNA and mitochondrial DNA than the black spruce [10, 66]. Furthermore, the genetic variation of the red spruce has no unique mitochondrial haplotypes, only subsets of those in the black spruce, convincing that the red spruce speciated peripatrically from the black spruce population that happened on the continent of North America [67, 68]. Based on these reasonings, we can reasonably conclude that *T. fargesii* var. *yunnanensis* is the product of a peripatric speciation event following a colonization event from central China that has seldom been reported in the HDM.

In addition to geographic isolation, natural selection has always been considered a key component of adaptive divergence and speciation [4, 69]. In this study, significantly ecological differences were detected for the two varieties (Figs. 5 and 6), which means that ecologically based divergent selection has played a crucial role in the differentiation of *T. fargesii* var. *yunnanensis*. Since the late Pliocene, the HDM has experienced strong orogenic activities and climatic changes, creating extremely diverse environments [17, 18, 47]. Such habitat alternation may have favored speciation through both geographic isolation and divergent selection [18]. Recently, dozens of candidate genes in plant species related to breathing, metabolism, and DNA repairing pathways have been implicated in allopatric speciation by population genomic studies [27, 70], underpinning the roles of natural selection in allopatric speciation in the HDM. Overall, these findings indicate that a combination of geographical isolation and ecologically driven natural selection has contributed the species diversification in the HDM.

## Conclusions

The speciation history of *T. fargesii* var. *yunnanensis* and *T. fargesii* var. *fargesii* was inferred using three coalescent-based population genetic approaches (DIYABC, fastsimcoal2, and IMa2) and niche modelling in this study. The results showed that *T. fargesii* var. *yunnanensis* and *T. fargesii* var. *fargesii* are a recent progenitor-derivative species pair. The formation of *T. fargesii* var. *yunnanensis* may be a consequence of geographical isolation following a colonization of *T. fargesii* var. *fargesii* into the HDM, coupled with ecologically driven natural selection due to the strong uplift of the HDM. Weak gene flow between the two varieties only detected in later stages of speciation, which is caused by second contact due to range expansions. Our findings demonstrate that peripatric speciation following the dispersal into the HDM may be an underestimated evolutionary scenario underlying the high plant species richness in this region.

## Materials and methods

### Population sampling and DNA extraction

Foliar samples of 112 individuals from 10 populations of *T. fargesii* var. *fargesii* and 63 individuals from 6 populations of *T. fargesii* var. *yunnanensis* were collected across their entire geographical distributions (Fig. 1, Additional file 1: Table S2). Fresh leaves were desiccated and preserved in silica gel after collection, and the voucher specimens were deposited in the Herbarium of Lanzhou University (LZU), China after the formal identification by Dr. Yujin Wang (Lanzhou University) (Additional file 1: Table S2). Total genomic DNA was extracted from approximate 20 mg of desiccated leaves of each individual using a modified CTAB procedure. In addition, two samples of *T. taxifolia* and one *T. grandis* from a previous study [50] were used as outgroups.

### Loci screening, amplification and sequencing

Because the mutation rate of conifers’ chloroplast genomes is very low (e.g., [50, 71, 72]), two chloroplast (cp.) DNA regions each with at least two variable sites, *trn*L-*trn*F and *rpo*B-*trn*C, were selected, amplified, and sequenced with previously reported primers [73]. In addition, 14 low-copy nuclear genes (*T8*, *T26*, *T82*, *T140*, *T147*, *T161*, *T173*, *T203*, *T212*, *T222*, *T235*, *T249*, *T275* and *T293*) were developed from transcriptome sequences of *T. grandis* (Additional file 1: Table S16) [74], a closely related species with *T. fargesii*. These nuclear loci had moderate nucleotide polymorphisms within and among populations (see Results) and were classified into various functional categories against four protein databases (Nr, GO, KO and Swiss-Prot), with the exception of *T26*, *T28*, *T161* and *T203*, which had unknown function (Additional file 1: Table S17). The primer pairs were designed using Primer 3 [75], with PCR product size ranging from 250 to 800 bp, GC content between 40% and 60%, primer length ranging from 18 to 25 bp, and melting temperature between 55 and 65 °C.

Polymerase chain reaction (PCR) was performed in a 20 µL volume containing 20–40 ng genomic DNA, 2× Taq PCR MasterMix (Tiangen, Beijing, China), 5 μm of each primer, and double distilled H_2_O. The PCR procedures included an initial denaturation for 4 min at 94 °C, followed by 35 cycles of 35 s at 94 °C, 30 s at 55–59 °C and 1 min at 72 °C, ending with a final extension of 7 min at 72 °C. The different annealing temperatures were chosen according to each primer’s features, 58 °C for *trn*L-*trn*F, 57 °C for *rpo*B-*trn*C, and 55–59 °C for nuclear genes (detailed in Additional file 1: Table S18). PCR products were sequenced with the PCR primers by the service of Sangon Biotech (Shanghai, China). Sequences of the same locus were aligned and checked using MEGA X [76]. All obtained sequences were deposited in GenBank database under accession numbers OP393246–OP393468.

### Genetic diversity and neutrality tests

Because chloroplast genome inherits uniparentally (either maternally or paternally) in the gymnosperms [77], the cpDNA regions were concatenated into a single matrix in subsequent analyses. The number of haplotypes (*N*_h_), haplotype diversity (*H*_d_) and nucleotide diversity (*π*, [78]) were calculated in DnaSP 5.10 [79]. Tajima’s *D* [80] and Fu’s *F*_S_ [81] were calculated using Arlequin 3.5 [82].

Nuclear sequences were firstly assigned to coding and non-coding regions by aligning sequences against their corresponding transcriptome sequences (Additional file 1: Table S16). For each nuclear locus, the population genetic parameters were estimated after phasing sequences using PHASE algorithm in DnaSP 5.10 with default parameters. We estimated the number of segregating sites (*S*), the number of haplotypes (*N*_h_), haplotype diversity (*H*_d_), nucleotide diversity (*π*), and Watterson’s parameter (*θ*_w_, [83]) and the minimum number of recombinant events (*R*_m_) in DnaSP 5.10 [79].

The expectation of neutral evolution was inferred for each locus using Tajima’s *D*, Fu and Li’s D* and F* [84], and Fay and Wu’s *H* [85] tests. These parameters are expected to approach zero under neutrality by comparing the observed value of the summary statistics with their expected distribution. In addition, the likelihood of natural selection acting on individual locus was estimated using the maximum frequency of derived mutations (MFDM) test [86], and the fit of data to neutral equilibrium was assessed by the multilocus Hudson-Kreitman-Aguadé (HKA, [87]) test. *T. grandis* was used as outgroup in Fay and Wu’s *H*, HKA and MFDM tests.

### Analyses of population genetic structure

Genetic differentiation between *T. fargesii* var. *fargesii* and *T. fargesii* var. *yunnanensis* was estimated using Wright’s fixation index (*F*_ST_, [88]). *F*_ST_ values for each nuclear locus and across all nuclear loci were calculated using AMOVA in Arlequin 3.5. The significance of *F*_ST_ was tested based on 10,000 permutations [89]. In addition, the relationships of haplotypes for each locus were constructed using a median-joining network implemented in Network 5.0 (available at http://www.fluxus-engineering.com, [90]).

The interspecific relationship was inferred by phylogenetic analyses based on the concatenated nuclear data from all individuals with *T. taxifolia* as outgroup. The maximum likelihood (ML), Neighbour-Joining (NJ) and Bayesian phylogenetic tree were constructed using PhyML 3.0 [91] with GTR model, MEGA X [76] and MrBayes 3.2 [92] with default parameters, respectively. The bootstrap support was calculated with 1,000 replicates. In addition, the phylogenetic tree based on the partitioned nuclear data was also inferred using BEAST 2.7.1 [93], with a relaxed-clock model and Yule speciation process. Two independent Markov chain Monte Carlo (MCMC) chains were run for 20,000,000 generations each, sampling every 1000 generations. The substitution model of sequence evolution in phylogenetic analyses was selected by jModelTest 2.1.10 [94].

Population structure with the admixture model was inferred by STRUCTURE 2.3.4 [95] using the dataset of 14 nuclear loci. Segregating sites in significant linkage disequilibrium after Bonferroni correction were excluded from this analysis. The number of clusters (*K*), varying from 1 to 8, was explored using 20 independent runs per *K*. Burn-in was set to 20,000 and MCMC run length to 200,000. The most likely number of clusters was estimated using Ln*P*(*D*) [96] and Δ*K* statistics [97]. The population clusters were visualized using the program DISTRUCT 1.1 [98].

### Inferences of divergence and demographic history

Because cpDNA variation in the two varieties is extremely low (only one indel and five substitutions in two chloroplast regions, *trn*L-*trn*F (901 bp) and *rpo*B-*trn*C (688 bp) were identified; see details in Results), we excluded cpDNA in subsequent analyses. The divergence and demographic history of two varieties was explored using approximate Bayesian computation (ABC) approach based on nuclear data. According to the analyses of genetic diversity, population structure and neutrality test (see details in Results), four basic scenarios (A1, B1, C1 and D1 in Additional file 1: Fig. S5) were formulated and modeled by DIYABC 2.1.0 [99]: (A1) *T. fargesii* var. *yunnanensis* derived from a colonizing population of *T. fargesii* var. *fargesii* at the time *t*_3_, then the population of *T. fargesii* var. *yunnanensis* began to recover from the founder event at the time *t*_1_, and (B1) a recent expansion event also occurred in *T. fargesii* var. *fargesii* at the time *t*_0_; (C1) *T. fargesii* var. *yunnanensis* and *T. fargesii* var. *fargesii* split from an ancestral population at the time *t*_3_, then *T. fargesii* var. *yunnanensis* experienced a bottleneck and a recovery during the time *t*_2_ and *t*_1_, respectively, and (D1) a recent expansion event occurred in *T. fargesii* var. *fargesii* at the time *t*_0_. Then, 20 sophisticated scenarios that were derived from the four basic scenarios were further simulated using fastsimcoal2 [34] with different migration parameters (Additional file 1: Fig. S5): (1) no migration in divergence history of *T. fargesii* var. *yunnanensis* and *T. fargesii* var. *fargesii* (A1, B1, C1 and D1); (2) initial migration occurred during the early phase of their divergence (A2, B2, C2 and D2); (3) recent migration occurred during the recent expansion of *T. fargesii* var. *fargesii* (A3, B3, C3 and D3); (4) initial migration and recent migration (A4, B4, C4 and D4); (5) ongoing migration occurred throughout the whole divergence history (A5, B5, C5 and D5). Therefore, of these scenarios, A1, A3, B1 and B3 conform to the expectations of peripatric speciation, C1, C3, D1 and D3 to the expectations of vicariant speciation, A2, A4, B2 and B4 to the expectations of parapatric speciation, and others to be complex event. The priors of all parameters were set with a uniform distribution (Additional file 1: Table S19). The generation time of 25 years, being applied to *Taxus wallichiana* in the same family Taxaceae [25], was used to estimate the demographic history of *T. fargesii*.

In DIYABC modeling, all one-sample and two-sample summary statistics were used to compare observed and simulated datasets. To ensure statistically robust results, at least 2,000,000 simulated datasets were generated for each scenario. The 1% of the simulated datasets closest to the observed data was used to estimate the relative posterior probability by logistic regression and posterior parameter distributions with 95% confidence intervals (CIs). The scenario adequacy was further evaluated through the fitting degree of priors and observed datasets and the level of confidence (including *type I error* and *type II error*) of the optimum scenario. In fastsimcoal2 simulation, the 2D-SFS (joint site frequency spectra) as input file was generated by Arlequin 3.5. The global maximum-likelihood estimates for demographic parameters were obtained from 100 independent runs with 100,000 coalescent simulations and 20 loops of the likelihood maximization algorithm. The relative fit of each scenario was assessed with the likelihood value and Akaike information criterion (AIC), and 95% confidence intervals (CIs) for the parameters were estimated by running 100 bootstrap SFS. The mutation rate of 5.58 × 10^− 9^ per site per generation (see below) was used to scale the demographic parameters. To verify the demographic inference of the above simulations, the Bayesian skyline plots in BEAST 2.7.1 [93] was used to infer the temporal changes in the effective population sizes of the two varieties based on the 14 nuclear loci.

The divergence and demographic history were further estimated using the IM model [33] based on nuclear data. The model assumed neutrality, no selection, no recombination within loci, and random mating in ancestral and descendant populations [100, 101]. After extracting the longest non-recombining region of each locus using the Imgc program [102], the demographic parameters, migration rate (*m*) and divergence time (*t*) between *T. fargesii* var. *fargesii* and *T. fargesii* var. *yunnanensis*, and effective population size (*θ*), were simulated and estimated using MCMC implemented in the IMa2 software package [33]. The simulations ran with a burn-in of 5,000,000 steps and retaining 2,000,000 steps under the HKY mutation model. The demographic parameters from the IM model are scaled by a mean mutation rate. The mutation rate was estimated according to *µ* = *K*_S_/2*T*, where *K*_S_ is the average divergence at silent sites between *T. fargesii* and its closest extant relative *T. taxifolia* endemic to eastern North America, and *T* is the divergence time between *T. taxifolia* and *T. fargesii* obtained from an estimate for the intercontinental disjunction (16.70 ± 3.0 Ma, [103]). The resulting geometric average mutation rate, 2.23 × 10^− 10^ per site per year, was used to scale the effective population size and divergence time. The mutation rate is comparable with estimates in *Pseudotaxus chienii* (3.44 × 10^− 10^ per site per year, [72]), a species in the same family Taxaceae with *T. fargesii*. We noticed that there was a much older time estimate for the age of the crown *Torreya* (43.3 Ma, [51]), however, their time estimate is not compatible with others for *Torreya* based on molecular dating (e.g., [103, 104]). In addition, the estimate resulted in an exceptionally low mutation rate (8.76 × 10^− 11^ per site per year) for the nuclear loci of this study relative to other conifers (e.g., 2.23–3.42 × 10^− 10^ per site per year for *Picea*, [105]; 7.00–13.10 × 10^− 10^ for *Pinus*, [106]; and 1.94 × 10^− 10^ for *Juniperus*, [58]). Therefore, we did not adopt the time estimate of Zhou et al. [51].

### Ecological niche modeling and ecological divergence

To estimate the effect of environmental factors on population divergence, the potential distributions of *T. fargesii* var. *fargesii* and *T. fargesii* var. *yunnanensis* were projected under the present-day (1970–2000), the Mid-Holocene (MH, ~ 6,000 years before present), the Last Glacial Maximum (LGM, ~ 22,000), and the Last Interglacial (LIG, ~ 120,000–140,000) climatic periods. A total of 75 sampled records (Additional file 1: Table S20), 16 recorded in this study and 59 retrieved from Chinese Virtual Herbarium (https://www.cvh.ac.cn) and the field investigation in the previous studies [44, 45], were used as input information. Twenty ecological variables (altitude plus 19 climatic variables) for each period, the present-day, MH (MIROC and CCSM models), LGM (MIROC and CCSM models) and LIG (MIROC), were downloaded and compiled from WorldClim database with a resolution of 2.5 min (https://www.worldclim.org, [107]) for each environmental layer. To minimize over-fitting of niche models, six variables (Annual Mean Temperature, Mean Diurnal Range, Temperature Seasonality, Mean Temperature of Driest Quarter, Precipitation of Wettest Month, and Precipitation of Driest Month) with pairwise Pearson correlation coefficients of *r* ≤ 0.70 were used to construct the species distribution (Additional file 1: Table S21).

The climate niches of *T. fargesii* var. *fargesii* and *T. fargesii* var. *yunnanensis* were separately modeled using MAXENT 3.4.3 [108] with the default parameters, included 80% of species records for training and 20% for testing the model and ten replicates. Model accuracy was estimated by the area under the ROC curve (AUC). An AUC value above 0.7 was considered as a good model performance [109]. In addition, niche difference between *T. fargesii* var. *fargesii* and *T. fargesii* var. *yunnanensis* was measured using Schoener’s *D* and a standardized version of Hellinger distance (calculated as *I*) in ENMTools 1.4.4 [110, 111]. An identity test for building niche models was calculated based on a set of pseudoreplicates (100 replicates) generated from a random sampling of data records pooled for *T. fargesii* var. *fargesii* and *T. fargesii* var. *yunnanensis*. The observed measures of niche similarity between the two varieties were compared with the null distribution. In addition, the differences of each ecological variable between the two varieties were assessed using nonparametric Kruskal-Wallis test and showed in kernel density plots drew by ggplot2 in R 3.5.2.

### Electronic supplementary material

Below is the link to the electronic supplementary material.

Supplementary Material 1

## Data Availability

The data presented in this study are deposited in NCBI GenBank under accession numbers OP393246–OP393468.
