# Supplementary material for: Peripatric speciation within Torreya fargesii (Taxaceae) in the Hengduan Mountains inferred from multi-loci phylogeography
Source: BMC Ecol Evol. 2023 Dec 12;23:74. doi: 10.1186/s12862-023-02183-1 (PMC10714551; doi:10.1186/s12862-023-02183-1)
Supplement: Supplementary file 1 — Supplementary Material 1 [file 12862_2023_2183_MOESM1_ESM.pdf]

Supplementary Information

The online version contains supplementary material available at XXX.

Additional file 1: Table S1. Variable sites in seven haplotypes from two chloroplast DNA regions, *trn*L-*trn*F and *rpo*B-*trn*C. **Table S2.** Sampling information, the distribution of chloroplast haplotypes, and chloroplast genetic diversity of *T. fargesii* var. *fargesii* and *T. fargesii* var. *yunnanensis*. **Table S3.** Nucleotide diversity and haplotype diversity across 14 nuclear loci in *T. fargesii* var. *fargesii* and *T. fargesii* var. *yunnanensis*. **Table S4.** Neutrality test across 14 nuclear loci in *T. fargesii* var. *fargesii* and *T. fargesii* var. *yunnanensis*. **Table S5.** Genetic differentiation (F_{ST}) between *T. fargesii* var. *fargesii* and *T. fargesii* var. *yunnanensis* for each nuclear locus and across all loci. **Table S6.** Posterior probabilities of four basic scenarios (A1, B1, C1 and D1 in Figure S5) modeled by DIYABC based on the first dataset (14 nuclear loci). The scenario with highest posterior probability was shown in bold. **Table S7.** Posterior estimates of demographic parameters for the best scenario (B1 in Table S6) revealed by DIYABC based on the first dataset (14 nuclear loci). **Table S8.** Posterior probabilities of four basic scenarios (A1, B1, C1 and D1 in Figure S5) modeled by DIYABC based on the second dataset (12 nuclear loci). **Table S9.** Posterior estimates of demographic parameters for the best scenario (B1 in Table S8) revealed by DIYABC based on the second dataset (12 nuclear loci). **Table S10.** The maximum likelihood estimates for each scenario in Figure S5 calculated by fastsimcoal2 based on the first dataset (14 nuclear loci). The best fitting scenario was shown in bold. **Table S11.** Posterior estimates of demographic parameters for the best scenario (B3 in Table S10) simulated by fastsimcoal2 based on the first dataset (14 nuclear loci). **Table S12.** The maximum likelihood estimates for each scenario in Figure S5 calculated by fastsimcoal2 based on the second dataset (12 nuclear loci). The best fitting scenario was shown in bold. **Table S13.** Posterior estimates of demographic parameters for the best scenario (B1 in Table S12) simulated by fastsimcoal2 based on the second dataset (12 nuclear loci). **Table S14.** Maximum likelihood estimates (MLE) and 95% highest posterior density (HPD) intervals of demographic parameters estimated in IMa2 based on the first dataset (14 nuclear loci). **Table S15.** Maximum likelihood estimates (MLE) and 95% highest posterior density (HPD) intervals of demographic parameters estimated in IMa2 based on the second dataset (12 nuclear loci). **Table S16.** Transcriptome sequences from *T. grandis* used to develop primers in this study. **Table S17.** Function annotations for 14 transcriptome sequences against four protein databases (Nr, GO, KO and Swiss-Prot). **Table S18.** Primer sequences, annealing temperatures, PCR products sizes, and gene ID of 14 nuclear loci used in this study. **Table S19.** The prior distribution of parameters

for all scenarios in the simulations of DIYABC and fastsimcoal2. **Table S20.** The geographical records of *T. fargesii* var. *fargesii* and *T. fargesii* var. *yunnanensis* used in the ecological niche modeling. **Table S21.** Pairwise Pearson correlation coefficients (r) of 20 environmental variables. **Figure S1.** Bayesian tree (left) and Neighbor-joining (right) tree were constructed by the concatenated nuclear dataset using MrBayes and MEGA, respectively. Support values were showed above the branches. **Figure S2.** Phylogenetic trees were inferred using BEAST based on the partitioned nuclear datasets, the first dataset (14 nuclear loci) (left) and the second dataset (12 nuclear loci) (right). Posterior values of all clades are lower than 0.5. **Figure S3.** The most likely number of clusters (K) inferred with Ln$P(D)$ (left) and ΔK (right) statistics implemented in STRUCTURE. **Figure S4.** Posterior probability distributions of effective population size (θ), migration rate (m) and divergence time (t) between two varieties estimated separately using IM model based on the first dataset (14 nuclear loci) (left) and the second dataset (12 nuclear loci) (right). **Figure S5.** Four basic scenarios (A1, B1, C1 and D1) with different migration models for the divergence and demographic history of *T. fargesii* var. *fargesii* and *T. fargesii* var. *yunnanensis*. N_F and N_Y represent the current population sizes of *T. fargesii* var. *fargesii* and *T. fargesii* var. *yunnanensis*, and N_1 and N_2 represent the population sizes between ancestral population and current population of *T. fargesii* var. *yunnanensis* and *T. fargesii* var. *fargesii*, respectively. N_A represents the ancestral population size. t_0, t_1, and t_2 represent the time of population changes and t_3 the divergent time. A2–A5, B2–B5, C2–C5, and D2–D5 are the derivatives of A1, B1, C1, and D1 by adding migration parameters at different times, respectively. **Figure S6.** Posterior probabilities for four basic scenarios (A1, B1, C1 and D1 in Figure S5) (A), model checking for the optimum scenario B1 (B), and level of confidence in scenario choice (including *type I error* and *type II error*) (C) estimated using direct approach and logistic regression in DIYABC based on the first dataset (14 nuclear loci). **Figure S7.** Posterior probabilities for four basic scenarios (A1, B1, C1 and D1 in Figure S5) (A), model checking for the optimum scenario B1 (B), and level of confidence in scenario choice (including *type I error* and *type II error*) (C) estimated using direct approach and logistic regression in DIYABC based on the second dataset (12 nuclear loci). **Figure S8.** Bayesian skyline plot inferred for *T. fargesii* var. *fargesii* and *T. fargesii* var. *yunnanensis* in BEAST based on 14 nuclear loci. The bold and thin lines are the median posterior and 95% highest posterior densities of effective population size through time, respectively. The effective population size and time were not scaled using mutation rate. **Figure S9.** The ROC curve (AUC) for each predicted distribution, present-day, Mid-Holocene (MH, under MIROC and CCSM models), the Last Glacial Maximum (LGM, under MIROC and CCSM models), and the Last Interglacial (LIG) climatic periods. **Figure S10.** Climate niches during Mid-Holocene

(MH) and the Last Glacial Maximum (LGM) under CCSM model were modeled and drawn using MAXENT 3.4.3 for *T. fargesii* var. *fargesii* and *T. fargesii* var. *yunnanensis*.

Table S1 Variable sites in seven haplotypes from two chloroplast DNA regions, *trn*L-*trn*F and *rpo*B-*trn*C.

	*trn*L-*trn*F (901 bp)				*rpo*B-*trn*C (688 bp)	
Haplotype	272	659	679	818	77	337
H1	-	G	C	G	G	G
H2	-	G	C	A	G	G
H3	-	G	A	A	G	G
H4	-	G	C	A	A	G
H5	-	T	C	A	G	G
H6	T	G	C	A	G	G
H7	-	G	C	A	G	T

-, missing nucleotides.

Table S2 Sampling information, the distribution of chloroplast haplotypes, and chloroplast genetic diversity of *T. fargesii* var. *fargesii* and *T. fargesii* var. *yunnanensis*.

Population	Location	Longitude (ºE)	Latitude (ºN)	Altitude (m)	*N*s	H1	H2	H3	H4	H5	H6	H7	H_d (SD)	π (SD)	Voucher specimen No.
var. *fargesii*															
1	Jinzhai, Anhui	115.76670	31.17065	589	7	0	7	0	0	0	0	0	0.000 (0.000)	0.00000 (0.00000)	LZU20140509
2	Zhangjiajie, Hunan	110.47444	29.05083	1420	7	0	6	1	0	0	0	0	0.286 (0.196)	0.00018 (0.00012)	LZU20150828
3	Shimen, Hunan	110.78158	30.09833	480	13	0	9	0	4	0	0	0	0.462 (0.110)	0.00029 (0.00007)	LZU20150807
4	Shennongjia, Hubei	110.38167	31.47361	1367	15	0	11	4	0	0	0	0	0.419 (0.113)	0.00026 (0.00007)	LZU20150813
5	Xunyang, Shanxi	109.42611	33.01778	699	7	0	7	0	0	0	0	0	0.000 (0.000)	0.00000 (0.00000)	LZU20150814
6	Chengkou, Chongqing	108.39778	31.96306	1138	16	0	1	1	7	6	1	0	0.700 (0.074)	0.00072 (0.00008)	LZU20150815
7	Nanchuan, Chongqing	107.13861	28.95056	1669	9	0	9	0	0	0	0	0	0.000 (0.000)	0.00000 (0.00000)	LZU20150826
8	Lueyang, Shanxi	106.32778	33.25583	989	16	0	16	0	0	0	0	0	0.000 (0.000)	0.00000 (0.00000)	LZU20150819
9	Wudu, Gansu	105.28111	33.02500	1556	11	0	9	0	0	0	0	2	0.327 (0.153)	0.00021 (0.00010)	LZU20150820
10	Baoxing, Sichuan	102.57750	30.61611	1864	11	0	11	0	0	0	0	0	0.000 (0.000)	0.00000 (0.00000)	LZU20150822
Total					112	0	86	6	11	6	1	2	0.398 (0.056)	0.00026 (0.00004)	
var. *yunnanensis*															
11	Lijiang, Yunnan	99.521564	27.246063	2401	6	6	0	0	0	0	0	0	0.000 (0.000)	0.00000 (0.00000)	LZU20160811
12	Diqing, Yunnan	99.486041	27.800994	2118	14	13	1	0	0	0	0	0	0.143 (0.119)	0.00009 (0.00007)	LZU20160812
13	Weixi, Yunnan	99.260547	27.160272	2648	11	5	6	0	0	0	0	0	0.546 (0.072)	0.00034 (0.00005)	LZU20160813
14	Weixi, Yunnan	99.012301	27.505637	1790	11	9	2	0	0	0	0	0	0.327	0.00021	LZU20160814

													(0.153)	(0.00010)	
15	Weixi, Yunnan	99.150355	27.077322	2110	11	9	2	0	0	0	0	0	0.327 (0.153)	0.00021 (0.00010)	LZU20160815
16	Gongshan, Yunnan	98.593004	28.091846	2389	10	1	9	0	0	0	0	0	0.200 (0.154)	0.00013 (0.00010)	LZU20160818
Total					63	43	20	0	0	0	0	0	0.440 (0.044)	0.00028 (0.00003)	

N_s, numbers of individual; H_d, haplotype diversity; π, nucleotide diversity; SD, standard deviation.

Table S3 Nucleotide diversity and haplotype diversity across 14 nuclear loci in *T. fargesii* var. *fargesii* and *T. fargesii* var. *yunnanensis*.

		Total						Nonsynonymous sites		Silent sites		Haplotype diversity	
Variety	Locus	*N*	*L*	*S*	π_t (SD)	θ_{wt} (SD)	R_m	π_a	θ_{wa}	π_s	θ_{ws}	N_h	H_d (SD)
var.*fargesii*	T8	112	786	29 (12)	0.00195 (0.00014)	0.00616 (0.00114)	1	0.00150	0.00684	0.00210	0.00623	29	0.731 (0.027)
	T26	112	290	4 (1)	0.00195 (0.00014)	0.00230 (0.00115)	0	0.00031	0.00148	0.00786	0.00530	5	0.512 (0.029)
	T82	112	288	3 (2)	0.00149 (0.00011)	0.00174 (0.00100)	0	0.00190	0.00223	0.00000	0.00000	4	0.415 (0.027)
	T140	112	333	2 (1)	0.00008 (0.00005)	0.00100 (0.00071)	0	0.00004	0.00066	0.00022	0.00211	3	0.027 (0.015)
	T147	112	681	28 (10)	0.00191 (0.00014)	0.00690 (0.00130)	1	0.00081	0.00362	0.00221	0.00810	31	0.725 (0.028)
	T161	112	388	19 (6)	0.00426 (0.00019)	0.00818 (0.00188)	1	0.00452	0.00812	0.00331	0.00851	19	0.794 (0.018)
	T173	112	417	5 (3)	0.00073 (0.00009)	0.00200 (0.00090)	0	0.00090	0.00249	0.00000	0.00000	6	0.296 (0.035)
	T203	112	410	32 (9)	0.00454 (0.00027)	0.01317 (0.00233)	1	0.00498	n.a.	0.00338	n.a.	38	0.902 (0.021)
	T212	112	448	3 (0)	0.00116 (0.00008)	0.00112 (0.00065)	0	0.00144	0.00140	0.00000	0.00000	4	0.483 (0.028)
	T222	112	407	6 (3)	0.00030 (0.00030)	0.00246 (0.00101)	0	0.00039	0.00280	0.00008	0.00156	6	0.087 (0.026)
	T235	112	379	6 (4)	0.00026 (0.00007)	0.00264 (0.00108)	0	0.00027	0.00228	0.00021	0.00396	7	0.096 (0.027)
	T249	112	420	20 (7)	0.00894 (0.00024)	0.00795 (0.00178)	3	0.01030	0.00848	0.00484	0.00638	20	0.680 (0.022)
	T275	112	405	3 (1)	0.00030 (0.00007)	0.00124 (0.00071)	0	0.00036	0.00108	0.00009	0.00174	4	0.120 (0.029)
	T293	112	427	14 (3)	0.00305 (0.00015)	0.00548 (0.00146)	0	0.00297	0.00409	0.00335	0.01008	15	0.795 (0.015)
	Average	112	434.14	12.43	0.00221	0.00450	0.50	0.00219	0.00351	0.00198	0.00415	13.64	0.476
var. *yunnanensis*	T8	63	786	5 (0)	0.00074 (0.00019)	0.00118 (0.00053)	1	0.00115	0.00189	0.00061	0.00125	7	0.236 (0.050)
	T26	63	290	0 (0)	0.00000 (0.00000)	0.00000 (0.00000)	0	0.00000	0.00000	0.00000	0.00000	1	0.000 (0.000)
	T82	63	288	0 (0)	0.00000	0.00000	0	0.00000	0.00000	0.00000	0.00000	1	0.000

				(0.00000)	(0.00000)							(0.000)
T140	63	333	0 (0)	0.00000 (0.00000)	0.00000 (0.00000)	0	0.00000	0.00000	0.00000	0.00000	1	0.000 (0.000)
T147	63	681	2 (0)	0.00148 (0.00002)	0.00054 (0.00038)	0	0.00000	0.00000	0.00186	0.00069	2	0.503 (0.007)
T161	63	388	4 (0)	0.00082 (0.00024)	0.00191 (0.00095)	0	0.00104	0.00240	0.00000	0.00000	3	0.191 (0.045)
T173	63	417	0 (0)	0.00000 (0.00000)	0.00000 (0.00000)	0	0.00000	0.00000	0.00000	0.00000	1	0.000 (0.000)
T203	63	408	2 (1)	0.00004 (0.00004)	0.00045 (0.00045)	0	0.00005	0.00059	0.00000	0.00000	3	0.512 (0.016)
T212	63	448	1 (0)	0.00007 (0.00005)	0.00041 (0.00041)	0	0.00009	0.00052	0.00000	0.00000	2	0.031 (0.022)
T222	63	407	9 (4)	0.00403 (0.00025)	0.00409 (0.00136)	0	0.00475	0.00496	0.00210	0.00173	7	0.755 (0.024)
T235	63	379	1 (1)	0.00004 (0.00004)	0.00049 (0.00049)	0	0.00005	0.00063	0.00000	0.00000	2	0.016 (0.016)
T249	63	420	1 (0)	0.00093 (0.00009)	0.00044 (0.00044)	0	0.00000	0.00000	0.00369	0.00175	2	0.390 (0.038)
T275	63	405	1 (1)	0.00004 (0.00004)	0.00046 (0.00046)	0	0.00005	0.00060	0.00000	0.00000	2	0.016 (0.016)
T293	63	427	4 (0)	0.00198 (0.00020)	0.00173 (0.00087)	0	0.00259	0.00226	0.00000	0.00000	5	0.598 (0.035)
Average	63	434.00	2.21	0.00073	0.00087	0.07	0.00070	0.00103	0.00059	0.00039	2.86	0.233

N, number of individuals; *L*, sequence length of each locus; *S*, number of segregating sites; π_t, π_a, and π_s, nucleotide diversity for all sites, nonsynonymous sites and silent sites, respectively; θ_{wt}, θ_{wa}, and θ_{ws}, Watterson's parameter for all sites, nonsynonymous sites and silent sites, respectively; N_h, number of haplotypes; H_d, Nei's haplotype diversity; R_m, minimum number of recombinant events; SD, standard deviation.

Table S4 Neutrality test across 14 nuclear loci in *T. fargesii* var. *fargesii* and *T. fargesii* var. *yunnanensis*.

Variety	Locus	*D*	*D**	*F**	*H*	MFDM (*P* value)
var. *fargesii*	T8	-1.90826*	-3.32491*	-3.29506*	-0.91400	0.00897
	T26	-0.27018	-0.42916	-0.44666	0.42160	1.00000
	T82	-0.23045	-2.27388	-1.90289	-0.59721	0.57399
	T140	-1.23475	-1.24669	-1.46828	0.02651	1.00000
	T147	-2.00637*	-2.63685*	-2.86177*	-0.74295	0.04484
	T161	-1.25679	-1.50995	-1.69681	-0.24455	0.24215
	T173	-1.20307	-2.50231*	-2.45229*	0.25665	1.00000
	T203	-1.84728*	-2.15409	-2.43932*	1.64798	1.00000
	T212	0.05228	0.76478	0.63024	0.36283	1.00000
	T222	-1.75378	-2.09030	-2.35291*	0.12204	1.00000
	T235	-1.80662*	-3.13897*	-3.19287*	0.09601	1.00000
	T249	0.32888	-1.89493	-1.21714	0.86635	0.95964
	T275	-1.19362	-0.75455	-1.06379	0.11771	1.00000
	T293	-1.09895	-0.42119	-0.81520	0.12988	0.57399
	Average	-0.56146	-1.68664	-1.75534	0.11063	
var. *yunnanensis*	T8	-0.75249	1.01748	0.50891	-2.90743	0.12800
	T26	-	-	-	-	-
	T82	-	-	-	-	-
	T140	-	-	-	-	-
	T147	2.53493*	0.66798	1.46612	-0.00457	0.96000
	T161	-1.08316	0.92117	0.31661	0.30324	1.00000
	T173	-	-	-	-	-
	T203	-1.00217	-2.08964	-2.05558	0.01575	1.00000
	T212	-0.90954	0.47855	0.06631	0.03098	1.00000
	T222	-0.03186	-1.80624	-1.40762	-0.25244	0.67200
	T235	-1.00217	-2.08964	-2.05558	0.01575	1.00000
	T249	1.21489	0.47855	0.82063	-0.70857	0.52800
	T275	-1.00217	-2.08964	-2.05558	0.01575	1.00000
	T293	0.27584	0.92117	0.83838	-1.26730	0.11200
	Average	-0.17579	-0.35903	-0.35574	-0.47629	

D, Tajima's *D* statistic; *D** and *F**, Fu and Li's *D** and Fu and Li's *F**; *H*, Fay and Wu's *H*; MFDM, maximum frequency of derived mutations test; -, failed to be computed for lack of enough variation. *, significant level at $P < 0.05$.

Table S5 Genetic differentiation (F_{ST}) between *T. fargesii* var. *fargesii* and *T. fargesii* var. *yunnanensis* for each nuclear locus and across all loci.

Locus	F_{ST}
T8	0.70245***
T26	0.14362**
T82	0.84377***
T140	-0.00352
T147	0.33467***
T161	0.12482***
T173	0.83991***
T203	0.11561***
T212	0.74915***
T222	0.46336***
T235	0.97766***
T249	0.42038***
T275	0.95762***
T293	0.18098***
Total	0.57650***

** and ***, significant level at $P < 0.01$ and $P < 0.001$, respectively.

Table S6 Posterior probabilities of four basic scenarios (A1, B1, C1 and D1 in Figure S5) modeled by DIYABC based on the first dataset (14 nuclear loci). The scenario with highest posterior probability was shown in bold.

Method	Scenario	Posterior probability	95% Credibility interval
Direct approach	A1	0.0881	0.0000 – 0.2119
	**B1**	**0.5208**	**0.3019 – 0.7397**
	C1	0.0505	0.0000 – 0.1462
	D1	0.3405	0.1331 – 0.5479
Logistic approach	A1	0.0042	0.0000 – 0.0215
	**B1**	**0.5511**	**0.5297 – 0.5725**
	C1	0.0025	0.0000 – 0.0198
	D1	0.4422	0.4207 – 0.4637

Table S7 Posterior estimates of demographic parameters for the best scenario (B1 in Table S6) revealed by DIYABC based on the first dataset (14 nuclear loci).

Parameter	Mean	Median	Mode	95% CI
N_Y	2.45×10^4	2.43×10^4	2.37×10^4	$1.24 – 3.70 \times 10^4$
N_F	4.61×10^5	4.37×10^5	3.49×10^5	$2.41 – 7.53 \times 10^5$
N_1	1.37×10^4	1.38×10^4	1.54×10^4	$0.22 – 2.56 \times 10^4$
N_2	5.64×10^4	5.55×10^4	5.66×10^4	$2.43 – 9.27 \times 10^4$
N_A	3.24×10^4	2.75×10^4	1.19×10^4	$0.35 – 8.08 \times 10^4$
T_0 (years)	5.33×10^5	5.15×10^5	3.75×10^5	$1.65 – 9.33 \times 10^5$
T_1 (years)	7.90×10^5	8.15×10^5	1.05×10^6	$0.09 – 1.43 \times 10^6$
T_3 (years)	3.20×10^6	3.23×10^6	2.95×10^6	$1.58 – 4.73 \times 10^6$

Table S8 Posterior probabilities of four basic scenarios (A1, B1, C1 and D1 in Figure S5) modeled by DIYABC based on the second dataset (12 nuclear loci).

Method	Scenario	Posterior probability	95% Credibility interval
Direct approach	A1	0.1141	0.0000 – 0.3909
	**B1**	**0.5308**	**0.0936 – 0.9680**
	C1	0.0496	0.0000 – 0.2293
	D1	0.3055	0.0000 – 0.7086
Logistic approach	A1	0.0049	0.0000 – 0.0416
	**B1**	**0.5423**	**0.4984 – 0.5862**
	C1	0.0027	0.0000 – 0.0395
	D1	0.4501	0.4060 – 0.4943

The model with highest posterior probability was shown in bold font.

Table S9 Posterior estimates of demographic parameters for the best scenario (B1 in Table S8) revealed by DIYABC based on the second dataset (12 nuclear loci).

Parameter	Mean	Median	Mode	95% CI
N_Y	2.39×10^4	2.32×10^4	1.88×10^4	$1.07 – 3.78 \times 10^4$
N_F	4.15×10^5	3.78×10^5	3.12×10^5	$2.17 – 7.33 \times 10^5$
N_1	1.09×10^4	1.02×10^4	8.78×10^3	$0.12 – 2.23 \times 10^4$
N_2	5.55×10^4	5.39×10^4	5.26×10^4	$2.19 – 9.34 \times 10^4$
N_A	3.83×10^4	3.42×10^4	2.46×10^4	$0.51 – 8.60 \times 10^4$
T_0 (years)	4.50×10^5	4.10×10^5	2.13×10^5	$1.09 – 9.30 \times 10^5$
T_1 (years)	9.03×10^5	9.63×10^5	1.36×10^6	$0.14 – 1.46 \times 10^6$
T_3 (years)	3.70×10^6	3.80×10^6	4.20×10^6	$2.22 – 4.83 \times 10^6$

Table S10 The maximum likelihood estimates for each scenario in Figure S5 calculated by fastsimcoal2 based on the first dataset (14 nuclear loci). The best fitting scenario was shown in bold.

Scenario	Migration model	log10(likelihood)	AIC
A1	no migration	-696.809	1405.618
A2	Initial migration	-697.050	1412.100
A3	recent migration	-695.556	1409.112
A4	Initial and recent migration	-695.603	1415.206
A5	ongoing migration	-695.641	1407.282
B1	no migration	-692.406	1400.812
B2	Initial migration	-692.494	1406.988
**B3**	**recent migration**	**-689.021**	**1400.042**
B4	Initial and recent migration	-689.963	1407.926
B5	ongoing migration	-690.147	1400.294
C1	no migration	-696.695	1409.390
C2	Initial migration	-696.894	1415.788
C3	recent migration	-695.565	1413.130
C4	Initial and recent migration	-695.573	1419.146
C5	ongoing migration	-695.158	1410.316
D1	no migration	-692.522	1405.044
D2	Initial migration	-692.537	1411.074
D3	recent migration	-689.982	1405.964
D4	Initial and recent migration	-690.106	1412.212
D5	ongoing migration	-690.213	1404.426

AIC, Akaike information criterion.

Table S11 Posterior estimates of demographic parameters for the best scenario (B3 in Table S10) simulated by fastsimcoal2 based on the first dataset (14 nuclear loci).

Parameter	Mean	Median	95% CI
N_A	3.14×10^4	2.55×10^4	$2.65 – 3.62 \times 10^4$
N_Y	3.63×10^4	3.48×10^4	$3.38 – 3.87 \times 10^4$
N_F	5.17×10^5	5.00×10^5	$4.99 – 5.35 \times 10^5$
N_1	2.49×10^4	2.43×10^4	$2.27 – 2.70 \times 10^4$
N_2	8.87×10^4	8.54×10^4	$8.25 – 9.48 \times 10^4$
m_{YF}	8.37×10^{-7}	6.94×10^{-7}	$7.19 – 9.56 \times 10^{-7}$
m_{FY}	1.91×10^{-7}	1.04×10^{-7}	$1.07 – 2.75 \times 10^{-7}$
T_0 (years)	6.10×10^5	6.12×10^5	$5.76 – 6.43 \times 10^5$
T_1 (years)	6.51×10^5	5.58×10^5	$5.56 – 7.47 \times 10^5$
T_3 (years)	3.65×10^6	3.74×10^6	$3.51 – 3.80 \times 10^6$

Table S12 The maximum likelihood estimates for each scenario in Figure S5 calculated by fastsimcoal2 based on the second dataset (12 nuclear loci). The best fitting scenario was shown in bold.

Scenario	Migration model	log10(likelihood)	AIC
A1	no migration	-502.335	1016.670
A2	Initial migration	-502.282	1022.564
A3	recent migration	-502.136	1022.272
A4	Initial and recent migration	-502.163	1028.326
A5	ongoing migration	-502.126	1020.252
**B1**	**no migration**	**-498.052**	**1012.104**
B2	Initial migration	-498.083	1018.166
B3	recent migration	-498.116	1018.232
B4	Initial and recent migration	-498.185	1024.370
B5	ongoing migration	-498.269	1016.538
C1	no migration	-502.416	1020.832
C2	Initial migration	-502.225	1026.450
C3	recent migration	-502.075	1026.150
C4	Initial and recent migration	-502.118	1032.236
C5	ongoing migration	-502.162	1024.324
D1	no migration	-498.124	1016.248
D2	Initial migration	-498.121	1022.242
D3	recent migration	-498.205	1022.410
D4	Initial and recent migration	-498.215	1028.430
D5	ongoing migration	-498.276	1020.552

AIC, Akaike Information Criterion.

Table S13 Posterior estimates of demographic parameters for the best scenario (B1 in Table S12) simulated by fastsimcoal2 based on the second dataset (12 nuclear loci).

Parameter	Mean	Median	95% CI
N_A	4.10×10^4	2.77×10^4	$3.43 – 4.77 \times 10^4$
N_Y	5.05×10^4	4.55×10^4	$4.64 – 5.45 \times 10^4$
N_F	4.81×10^5	4.60×10^5	$4.55 – 5.07 \times 10^5$
N_1	1.76×10^4	1.71×10^4	$1.53 – 1.99 \times 10^4$
N_2	9.19×10^4	8.53×10^4	$8.54 – 9.84 \times 10^4$
T_0 (years)	4.98×10^5	4.79×10^5	$4.52 – 5.44 \times 10^5$
T_1 (years)	6.06×10^5	5.33×10^5	$5.26 – 6.86 \times 10^5$
T_3 (years)	3.46×10^6	3.49×10^6	$3.27 – 3.65 \times 10^6$

Table S14 Maximum likelihood estimates (MLE) and 95% highest posterior density (HPD) intervals of demographic parameters estimated in IMa2 based on the first dataset (14 nuclear loci).

	θ_F	θ_Y	θ_A	$m_{Y>F}$	$m_{F>Y}$	t	N_F	N_Y	N_A	2*Nm* (F)	2*Nm* (Y)	*T* (years)
MLE	2.5460	0.1427	0.3930	0.28700	0.01125	0.2335	272,784	15,289	42,107	0.00080	0.36535	2,501,768
HPD95Lo	1.9660	0.0888	0.1910	0.00300	0.00000	0.1475	210,641	9,514	20,464	0.00000	0.00295	1,580,346
HPD95Hi	3.2580	0.2347	0.7170	1.18100	0.09285	0.3165	349,069	25,146	76,821	0.01090	1.92385	3,391,047

θ_F and N_F, effective population size of *T. fargesii* var. *fargesii*; θ_Y and N_Y, effective population size of *T. fargesii* var. *yunnanensis*. $m_{Y>F}$ and $m_{F>Y}$, population migration rate from *T. fargesii* var. *yunnanensis* to *T. fargesii* var. *fargesii* and *T. fargesii* var. *fargesii* to *T. fargesii* var. *yunnanensis*; 2*Nm* (F) and 2*Nm* (Y), population migration rate for *T. fargesii* var. *fargesii* and *T. fargesii* var. *yunnanensis*; *t* and *T*, divergent time between *T. fargesii* var. *yunnanensis* and *T. fargesii* var. *fargesii*. θ, *m* and *t* are scaled by the mutation rate, while *N*, 2*Nm* and *T* are scaled by individuals or years.

Table S15 Maximum likelihood estimates (MLE) and 95% highest posterior density (HPD) intervals of demographic parameters estimated in IMa2 based on the second dataset (12 nuclear loci).

	θ_F	θ_Y	θ_A	$m_{Y>F}$	$m_{F>Y}$	t	N_F	N_Y	N_A	2*Nm* (F)	2*Nm* (Y)	*T* (years)
MLE	1.7820	0.1518	0.3430	0.12700	0.00025	0.2233	206,951	17,629	39,834	0.00002	0.11316	2,593,270
HPD95Lo	1.2860	0.0903	0.1010	0.00000	0.00000	0.1523	149,348	10,487	11,730	0.00000	0.00000	1,768,719
HPD95Hi	2.4020	0.3152	0.6870	0.63700	0.07875	0.3488	278,954	36,605	79,784	0.01241	0.76504	4,050,751

θ_F and N_F, effective population size of *T. fargesii* var. *fargesii*; θ_Y and N_Y, effective population size of *T. fargesii* var. *yunnanensis*. $m_{Y>F}$ and $m_{F>Y}$, population migration rate from *T. fargesii* var. *yunnanensis* to *T. fargesii* var. *fargesii* and *T. fargesii* var. *fargesii* to *T. fargesii* var. *yunnanensis*; 2*Nm* (F) and 2*Nm* (Y), population migration rate for *T. fargesii* var. *fargesii* and *T. fargesii* var. *yunnanensis*; *t* and *T*, divergent time between *T. fargesii* var. *yunnanensis* and *T. fargesii* var. *fargesii*. θ, *m* and *t* are scaled by the mutation rate, while *N*, 2*Nm* and *T* are scaled by individuals or years.

Table S16 Transcriptome sequences from *T. grandis* used to develop primers in this study.

Gene ID	Sequences
c23789_gl	AGCACGAGCACGAGCACGAGCACGGTTCGTTACACAGTGCATACGGTTTCAACAGTCCAAACATTGTCAACAGCCTGCTCGTTTTTCTGCTTAGAGTGCCGCTGTTCGTTTCAATCAACCGAAGCAAAAATGGCGCCGGATCACAAAACTC<br>TGGTTTTTGCAGATTCGTCTTTTGATGGCATTTCCGAGTCTTCTTCTTCCTGTTTATCTGACGATGGGCAAGGAGCAGGAGGGGGAGGGGTTGTGAGTTCTTGGTCCTGCAAGAGGACGTATGGGCTTGTGGCTGAGGATATAAGCAGCATG<br>GTCAAGAAGAGGAGGAAGAGAATGCTGGATGAGAATCTCTCCGTCTTGCGTTCCATTACACACTCTAATGCGACCAGGAAAAGCTGCATAATATCGGATGCTTTCAAGTATATTGAGGAATTGAAGCAGAGGGTCCGTGAATTGAATAGG<br>AATCTTGCGGCAGAGGTTGATGCCCGCCAGAGGGAGTTGCTGGGGTCCCATCCCCATGAGACAGGGAGAGTGAATTCAGGGCCTCCTCTGCCTATTGTCACAGTGGTGGGCAAGGAATGTGGGCTTGAGATCCATGTCTCCTGCGAAAAAT<br>GGCCAGGGTTGCTGATTGCTATACTGGAGGCTGTGGAAGCTCTGGGTCTCAATGTGCTTCAGGCCAGGGTGTCTTGCAAGGACTATTTTCTCTTTGAAGCTTTGAGCGGAGAGGACAAACAAGGTAAAATTGCAGATCCCCATATCGTTAA<br>AGGAGCTCTCCTGCAAGTCATAGACAGGATAAACCCAGAAAAGACTTCTGCAACAGGCAGTGAAGTTATGGACTAGAGATCCTGCAACAAGTCCAGGTTCAAGTCCCACTGGCCTGAGCCAAGATTTAAACCTGAGAATGAATGACCAGG<br>TCAATTCCAGACAAGGATCAAGTGGGTTTTGGTGAAATTATTTTTCAGAGAAGGAACTTGTACATGATGAAATGGTATTTATTATTATCCTATAAAGGAAGAAGACAGAGAGATGTAATGTTAGGAACCCTGCAATTGTATCAGTCTAGTT<br>ATATACCCTCTTTAATTTGGTTATATTAGAAGAGAGCCTAGGCCTACTCTGAAATTGTTGTGTAAAATCACAGTTGCTATGCACATGTTTAGAGCTGTAAGTCAGTCAAAGGTAAACAGTTCTACTCCTGTGCATTTATTACAGGCCACCCAT<br>TACAAGAATAATATAATATGTTACTTTCAACGAATTGTAATGTGTAAATTCCTTTCTGAAAAGGACTGGTGTGGG
c28921_g1	GGATCAGCTATCCTATCCATTATAGAAATAGAGGGGCGGGGCGCATAAATATCTAATCCTCCCATCTCCAATTCAACCAGTTACCAGTCACCAGTCACCAGTATAATAGATATGATACGAAATGGGATTTGTGAATGAGAGGCAGGAAGTG<br>GTTGCGACGTAGAAGCATTCTAAACAACTTAGACCTCTATAAACCGGTATCTCGACGGCGGCCATTGCACAATATTTAAAGGTACGGGAACTGCAATCCTGCACAATATAGAAATTAAAAATATCTCCTTTTTTCCTTACCCAAAGGGGCTC<br>TTATTTCTGCTCACCGCACCTCACCAAGGGCTTCCAAACCCCTATCGGAGCCTCACGAGATTATCGTGTAAGAGGTAGATTTGATTGCACAACTGTAAGATGATTATTCGCTTGTTTTTAAAGTTAGGGTTTTTTTATGGGTCGGGATCTGC<br>GTCTGTTATAAGACTCTTAAGATTTGGAGAGTTGTAGACAGTCTTGAGTAAAAGATGCAGGTTTTGCAGTATAGGCGAGGTGCGAAGGAAACGAAGAAAATTTTGGGGCTTCAACTGAGAAATGAGTCGGAGAGGTCTGTGTTGTATGATT<br>TTTTCTCTACTTCTTTCCATCTATGATTTTGTGTTGTTTTTACCATTTATTATGGTTTCAGGGTGTATCAAGTGTTGTAGATAAGGGCAACACGGTGGCTCCCCCAGTGGTGGCACCTATGGCCTCCCCACCATCACAACTGGCCCCTATAGCT<br>GTGTTTGTACATCTGTATTATCTGGACATCCATATTTTTCACCCGATGAGCATGCATTATATATGGTGTTATACATAATAACATATATATTATTTCGTTTATAGAGTGCAGCAGAAAATGCAAGGTGGGGTGCAAGAATGTTTAAGCTCACA<br>AAGATCGGTGCTTTAAATACTGCAATGATTGTTATATGGTGTGTAAATGTGTTCCCAGTGGGCA
c61083_g1	GGACAATCTCTTCTTCAATATATCATTGGGTTCTTTTAAAAGATTTCCTAGTAGTTCTCAAACATTCTCATGCTTACCCTGATAGATATTATAGATTTCAGCTATGATCTCACTATCATTTCTCAAGAGTCAAAATTCCTTCTCGAAAGCTTTC<br>CTCAATTTGTACCATCGACATTTATCAATGGCTAGGTCATTTGTTCCCTAATCAATGGCTACACCTTGAAGTGTCACCGGAAAACACCATACCCAAAACTGTTCACTAGAGTGCCCATTTGTTTCCCATATTGTAATACATGTATGAAAATGC<br>CATACGGGATCCTTTGATATATCCCTGATGACTTTCGGTAATTTTGCTTTTTTGGAGATGGAGTTGGTGGGGTACCTATTATTCTAGTTATGGGTTGCTTATGACTAGAATATTGAAATACGATTCTCCTCTCTGAGAAGGTCGGTGCTATAC<br>ATTCCTCTATACTCGGGCACCCGCCCCTATGTTCACCCCTTCCTTCAGTAGGGATGGTTCACTCTCTTGCCCTGCATCAAGTCCTTTCAATTATACTTTCTTCAATTTGTGGTTTGGCTGCTATGATGTGATTGGCTCTTTGTCATCAATCAATA<br>GCTATTCTATCCCGAGGATATTTGTTTTATACGTGATTCTCTGCTTTTGTCTCATACTTTGTTTCAAACCACAGACTTCCCTGGAAAAGAGGGTGCTCTATCAATCCTCACTAGATTCTATAGAATCCCTTGCAGACCTTACGAACTATATTCG<br>ATTGCCAAGATCAACCCTTCCTCTATATGTGGTAGTTGCGTTGCAACCTCCAACGATTGTGAACTTGTAAGTGGTGTTTGTTGCTCTAGCATTGTCTTATCCCTTCCTAGGTTTCATCTCACGCTTCATTTCGGCTTCAGCTTTCACTACTGTGC<br>ATGAAACAAATGTTAATGCTCCACACTCATCTTGATAATATTTTCCTAAGCATATAATTGACCAAAGAATTGGAAGAC
c32904_g1	AGAGGAAGAGGAAGAGGAAGAGGGCTTGGAAGAGGGCTTACGCATTTCCATTTCCATTTCCATTTCCATTTCTACTGCTTCTTCGTCCTTCTCCTAGTTGGTCTGCAACCTTATCTTAATGCAAATAATCCCCCTTCCTTCGTAGCTCTTCCA<br>ATTGCATTTTCCTCGGGTTTTAGGTCTACCCAATGGCATCTGCAATACTATCTTACCGTGGGTTATATACAAGTTCACCCAGTGGACCACCTTCACATAGGAGCCTGAAACACACACCCCTGGTCTCCTCCTCCCCTTTGTTACGCATTGGGA<br>ATGGGTGTTTCCTTAAACAAAAGGGTTATTTGCGCTATGATTCTTTGAACACAAAACATAATGTGGCGGCACCTCTGAGATCAACTTTGGTATCTCAAAATGAAGATGAAGAGGATGGAGAGGACAAGTTTGAGCCGAGCGCATCTCCACC<br>GTTTAATTTGGCAGAGATTAGAGCAGCCATTCCGAAGCATTGCTGGGAGAAGAACAGTTGGAAGTCACTGAGCTATGTGTTGAGGGATGTAGCGCTTGTTTTTGGTTTGGCAGCAGGAGCTGCACATTTCAACAATTGGGCATGCTGGCCC<br>ATTTATTGGATTGCCCAGGGAACAATGTTTTGGGCTCTTTTTGTTCTAGGCCACGATTGTGGCCATGGAAGCTTTTCCAACAACCAGAAATTCAACAGTCTGATCGGGCATTTGACACATTCTTCTATTTTGGTGCCTTATCATGGCTGGAGA<br>ATCAGTCATAGAACTCATCATCAAAACCACGGGCATGTGGAGAATGACGAATCATGGCATCCGATGTCTGAGAAGATTTACAACACTTTGGATGAACCAACAAAGAGGCTGCGGTTTAACATTCCTTGGCCCCTATTTGCATATCCTTTCT<br>ACCTGTGGGGCAGAAGTCCTGGGAAGAAAGGTTCCCATTTTCACCCTGACAGTGATCTGTTTGCCCCCAGTGAGAAGAAGGATGTTATTACATCAACCGTGTGCTGGACTGCAATGGTGGCTTTACTTGCAGGATTTACAGTTTTTATGGGT<br>CCATTACCGATGCTGAAGCTCTATTTTATTCCCTATTGGATTTTTGTAATGTGGTTAGATCTAGTAACATATCTGCATCATCATGGTTACGATGAGAAAGTTCCGTGGTACAGAGGCAAGGAATGGAGCTATCTTAGGGGAGGGCTTAGCAC<br>CATTGATCGAGACTATGGCTGGATTAACAACATCCACCACGATATTGGAACCCATGTTGTTCATCACTTGTTTCCTCAAATACCTCACTACCACTTGGTGGAAGCGACTGAAGCCATTAAGCCTGTGCTTGGAAGATACTACCGGGAGCCTA<br>AGCAATCTGGTCTTTTTCCGATTCACTTGCTGGAGCCTCTACTCAAAAGTATGGCAGAGGATCACTTTGTTAGCAACGAGGGAGATGTTGTCTTCTATCAAGCAGACCCCAACCTCAGAGACTAGTCACTAACTGGGCCCCTTCTTTGTATTT<br>TATTCTATAAATCTAGAATTTAATTTAATATCCTTTGGGCTTCAACCATTTCAAAAGGATGCAATACAATATAATTCTTCCAATTCTAGGTTTACTTGGTGGCACTCACTGTTACCACCATGCCACCATTCCATTTTTTTCTGTAATTTGAAAA<br>TGTGGGTTAATAGTAAATAGCAGCAAATGTTGGTGGAATATTTACAGATGGAGTAGTAGTAGTATAGTTTTAATTACAATTAAATCAAATCAGTATGGTTTGCCAATAAATTGATGAAGTGTGA
c33855_g2	AGAGCCTCACTACATAAGATGTGTGAAACCAAACAGTGTTCTAAAGCCAGGCATTTTTGAGAATTACAATGTTCTACAACAACTACGTTGTGGGGGTGTCATGGAAGCAATCAGAATCAGCTGTGCTGGGTATCCCACAAGACGAACTTTT<br>GATGAATTTCTTGATCGTTTTGGTATTCTTGCCCCTGAAGCATTGGATGTAAACTACGATGAGAAGACTGCAAATAAAATTTTACTGGAGAGAATGGGTCTCCAAGGCTATCAGATAGGGAAAACTAAGGTTTTCCTGAGAGCGGGGCAAA<br>TGGCAGAACTGGATGCTCGAAGAACCGAGGTATTAGGAAAAGCAGCAAAAATTATCCAACAGAAATTTCGGTCACATATTGCTCGAAAAGAATATCTTGCTTTGCGCAATGCGGCCATTCATTTGCAGGCTTTATGGAGAGGTAACCTTGC

TCGTGAGCTCTATAAAAACATGAGGAGGGAGGCTGCTGCAGTGCGGATACAAAAGCATGTGCGCAGATATCAAGCTCGCAAGGCTTACAAACAGTTGCAATTGTCAGCTGTTGTCATTCAAGCAGGTTTACGTGCAATGGCTGCAAGAAA
TGAATTTCGATTTAGGTCTCAAACTAAAGCTTCAATTATTATTCAGACTCAATGGCGTGCATATAGAGCTCGCTTATACTATAAGAAGCTAAAGATGGCAGCCATAACATTCCAGTGTGCATGGAGAGGAAGGGTTGCAAGGAAGGAGCTC
AAACAACTTAAGATGGCTGCTAAGGAGACTGGAGCACTGCAAGAAGCAAAATCCAAGTTGGAGAAGCAAGTAGAGGAGCTTACATGGCGCTTGCAGCTGGAGAAGCGTATGAGGGCTGATCTGGAAGAAGCAAAATCACAAGAGACTG
CAAAACTGCAATCTGCCCTACAGGAGATGGAAGCACAGGTTAGAGAAACTAACATCTTGTTGGAAAAAGAGCGAGAGGCAGCAAAAAAGGCTATGGAACAGGCTCAGACTGTAAAAGAGACTCCAGTCATCGTGGACACTGAAAAGCTC
GAGATTCTTTCCAGTGAGAATGAGAAGCTGAAGGCTTTGATCAGTTCACTTGAGAAAAAAGCAGATGAGGCTGAGAAAAAAGCAGATGAGGTTGAGAAGAAATATGTCGAATACCAAAAGCTGAGTGAAGAAAAGCTTAGAAAGGCAC
AGGAGGCAGAGTCAGAAATTGAGAAGCTCAAGGATACATTACAGAGGTTTGAAGCAAAACTTTTAAATGCCGAATCTGAGGATAAGGTCATTCGCCAGCAGACACTGGTCAAGTCACCTATCAGGGCCTTTTCGGAACGCTTAATGACAA
CAGTTGCTCAGACTCTTGAAAATGGCTATCATGAACCTGAGGAACTCAGTACTGTCAAAGTTACTGTTGCAAGCGAGGTACCTGTCTTCAAGGAGCATGCTGAAATTGAGAATAAGCCACCAAAGTCTATTAATGACATAGAGAATAAACC
ACCAAAACCTCTTAATGATATACATGAAAGCATAGACACACTTATCAAGTGTGTTTCACAAGATATTGGCTTCAGCCAAGGCAGGCCTGTTGCAGCATGCATTATTTACAAGACACTTTTTCATTGGCGGTCTTTTGAAGCTGAGAAGACCA
GTGTGTTTGATCGTATTATTCAGATGATTGGTTCTGCGATTGAGAATCAAGAAAATAATGATGTTTTGGCATACTGGTTGTCAAATACGTCAACTTTGCTTTTCTTCTTACAGCGTACACTGAAGGCAACTGGTGCAGCTGGAGTAACTCCCC
AAAAGCGAAGATCATCTTCAACTTTACTTGGAAGAATGGCTCAAGGTTTTCGGTCCTCACCATCGGCCATGGGCCTTCCATTTGGCATTGGTGGATTGGATGCTGTGCGCCAAGTGGAGGCAAAATATCCAGCTTTATTATTCAAGCAGCAA
CTTACAGCTTATGTTGAGAAGATTTATGGGATCATTCGTGATAATCTGAAGAAGGACATATCTGCCTTGCTTGGCCTGTGCATACAGGCACCGAGGACATCAAGGGCAAGTATGGTAAAGGCATCTCGATTGCAGGCAAATGCAAGTGCAC
AACTAGTTCCCAACCACTGGCAAAGCATTTGCAAGAGCCTAACCAATCTTCTCCAAACTATGCGTGCAAATTATGTGTCGCATGTTTTGGTCAATAAGATCCTCAACCAAATATTTGCGTCCATTAATGTACAACTTTTTAACAGCCTTCTTT
TACGCCGTGAATGCTGTTCCTTCAGCAATGGAGAGTATGTAAAGGCAGGGCTAGCTGAGCTGGAACTTTGGTGCTATGAAGCGAAAGAAGAGGCTGCAGGCTCGGCTTGGGATTCACTCAAGCATCTCAGACAAGCAGTGGGATTTTTGG
TTATACATCAGAAACCGAAGAAAACCCTGGATGAAATTACCCATGATCTTTGTCCGGTCTTAAGCATACAACAATTATATAGAATCAGTACCATGTACTGGGATGATAAGTACGGCACTCACAGTGTGAATCCAGAAGTTATTGCAAACAT
GAGGGTGTTGATGACAGAAGATTCAAATAGCTCTATTGGCAATTCTTTCTTGTTGGACGATGATTCAAGCATACCATTTTCTGTGGATGACATATCAAAGTCTATGCGAGAGACAGATTTATCAGATATCAATCCTCCTCCTCAGCTTCTTGA
AAATTCAGCATTTCAGTTTTTGTTAATAAATGCAGAGTAGTACGGAATGCTTCACTTTCTGATTCATTTTCGTCCCTTGATCAGTATCGTCATCCGTTCACCTGTGATTTCTATTTGTACTTCTAAAATGTTCTTGTCTCAGAGCAGCCAAGTA
TACCTGCGAGGAGAATAGTTTCCTACCCACTCGATTTGTTTCATTTTTTGATTACTATAGGGGATATGATTCTTATGTAATTATTTGGTTTTGGATTGTGGCTTTTCCCTTGAAGGCTTTATTTATTGTGGTCTGCAAGGGCAGGTTACCACAA
AAGTGTAAGTACAGAAGAGAAGTTCTTCTGAGCTTAAGTTTGCTTGCCTGTGAAAGTTTCCATACTTTCGGCAAGCTTAAGGTAAAAATGTTGTTTCTTCGTAGATGCGGCTCTATTTTGCTGCATTCAGTTACAAGGAAGATTCCTTAGCTT
TTGGTGTTGTGTTACCTGAAGGTTAGGGATGTATTTGGATTCAATCGTAGCATTCTTTTTAGTTACAGATATGTATACTCATCATTTGAAATTGATGGGAGCCACCATTGCGCCCTTGAATGTAATATTTATGCATTTATGCTGTTCGTTGTAT
AAAAGCCGAAAAAAAAAAGAATTTTGTAGTGTGTCCACCATTACCATGCTGAGACACAGGTTTGAGTGAACATTGATTAGGGTCCAGTCCTCTTTGAGTTGTGTTCTACAGTCAAAATATCCGGTTTATGGATAATGTAAATTCTGTTTTTG
TGGATATCAAAAACAAAATTAAAGCTTCTGTAAATAACATTTTTTCAGCTGTAGTGGTTATTTTTCAGACTCCCATTATTTCTGGGAGTGGACTTAGAGATTGTG
TTTACGAAAACAAATGGTCAAAACAAAATGGACCACAGCAGCAAATTTTCGGCTAGCAAACCCTAACATGATTTTCCCTGTAAAGCACCTATATAGCAATTACAACAAACAAGTTTTAGTATTCTGTAAAACATATCTTAATTTTCAAGAA
GAAAAAGTGGGGGACTGAACTAATTCAATACAGTTGAAAGCAATCATCAAATGGGATGAGCTTTAGCCATTAATCATTGGGATTTTGTAGAGCAACTCGAACCCCAGGTTAAAATCTCCGGGAGACAAGTTAAGAGTTAATATTGGGAAA
AATGATAAAGGTTGATTTTATGGTCACAGCCATGCCAGATGCCATTTACTTGATATATTGTTTAAACTTTTGACCCCTGGGTTTCAACCTTGTGATCAAGCTCAATAGTAAACCAGACCTTTTTAATCCACCTACACCCAGAGTGCAATTTAT
c61438_g1
CCAAAACTGGAATACCACATCCATCTCAAGGAGGTCAATGCCAACAGAAAAGGCCTGGTGCAAATCCCTAGTGACTTTGGCTAAAAAA
GATACATAAATATAATTCTCAAAACTTTTGTTTAAGAAAAAAACAGGACGAAGTGTCCCACAAATCTCACAACCATGAAAGTAAACACCATTTTCAAATGTACATGTTGGGCAGTTCCAAACCAACCCTAAAGTTTCACGATGCAAAGGCAA
CTCAGATGATCCAGTGATCTCAAACCTGGGGGTCTCACATACAGCACAAGCATATTGGTTATCTGCAATTGAATTGTCATATGTGCATGCATGACAACTCCAAACCATGTTTATAGTTTCATTAGGTTGCTCTGAATCTCTGGTCCTCGGGAT
CTTAAGTGGGGAACGGAGCCACCTTTGGTTCAAAAATAACCATGAACTCCAGTTGCTGAATAGCCAAGGTCGATAAGAATACAGAAAGCCTGATAGAGCACCACAAACAGGACCATAAAAAGGCATTATTGGACATAAGTAATAGAGAA
c32552_g2
AAGCAGTTTCAACTGCATAAATTGCTGAAAGAGTCACTCTATGGATAATGTGCTGGAAAGGGCCAGGTAGAATGGATCTATAAATTTCTGAGCTAGTGTTCAAAAGAAAGTTCATGGCAAAAAGTAACCCAGACATGCCAGAGTGACACA
TTGGCAGACATATGTCTGCTCCGGACAAATGTTTCCAGAACACTGCTGGTAAGATTAGTATCCCATCATAGGTGAACAATAAAGCTAAAATCAACAAT
AGAAACCATCTCTTCTACAGAATGCGCCACAAGTGGGGTGCAGAGAAGACAACCATTTTGAGCCACTGCTGCCATTGTCAATGTTAGACCATATATCAACGTGCAGTTGATTGCTGGCAACTTAATTATCTTGCTCAGATATTCATAAAACC
TAGAGCTATGTTTGAGATTTTAACGAAAGCAATGCTCCATATTTGGCAGTCGCTGGTAAGATACAAGATTAGCTAGAACCTACTACATTCACAGACAAATAATACAATGAAAACTTGCTCGCCAGTGATCTCAAAATCCACTAGTTCTACG
TTCCAAACAGGGAATTCTAATAGCTGATATATGGGACAGTCAAATCTTAGCTATATCTATGGAGTTTGGCAGCAGCACTCAGACAAATAGAGGTGACCCACAACTACAGACGCCTTTTTGCAATGCGGCTAAATACAGTTATGCGATCGAC
c23898_gl
CCCAGGTCTGACACGGCATGTAGATGTTGCCTCAACACCATCTTAACCCCTAAGTTGAGTTAATCTGAATAAAACAATGACCGGTTCCCGTGATGAATCCTTACCTATAACTGTCATTCCACTCTGTACCAAAAAATAAGTTTCGGAAATTA
ATTGCAAGAGTTGTGAAAAATATTGATAAGAAATATATTAAATACAATTATCGGACTACACACCAAAATCAAAACTTTGACCTAATTAAAACGGAGCAGGTGAAAAACATTTTGAGCAAGGCATCTGAGAGAGTGGCTAACAAAATCTTA
TGAGTATGTATAGCACACGAAATAACTCTAACATATAGGACATCATAAATATCAGACCAGAATGCCATGCATGATCTGTAAAGGGTGGCATTCACTTCTAATTAAATTGCTCGCTGTTTATTTCTCTTTAGCAGTATGGTCAGTTTATTAAC
CCTCCATTCCTTTTGTGCAGCAAACTTTGAAGTTTGGTCTCCACCAGGGGCATTCTTCTCTTATGTAAGGA
GCTCTAAAATGTTATGGAATGCCAAAGCAAGTAGGTCACTTAAGAGAGAGAATAAAAGGAAGGTGCAAGTAGATGCGGCCCAGACGATAAATCCTCTCACTTCCAAGGAATTTTCAATTCAAAAAACAAATAAAGCAACCCTTGAAAATT
c30526_gl
CCTCGTTAAGGTCCCAAAGCAGTAAGATTTGATCTGTAAGCTTTTTAACAGTCTTCCCGTTGCATTGAATGTTGCGTGGATCTCCTCTTGTTCGGTAATTTTCACTGGTTGGTCTCCACTCTCCAGCCAATCATAAGTTTTGATGGGGAACCAT
GAAACTGAGCTGTGCACTGTGGAGGTGGGAGATGGAATTAATCTTGTGACAATGTTATTTAAACCCACATCTGTGACAAATGATGTTGTCATTGTCTTGGTTCATCAATACTCTGTATTGGGTGGTTGCCAAGGACTCATGAAGGGTATTGC
TAACGAGTTAGCTGAAAGAGGTTATAGAATCGTGACTTTTGATATGAGGGGAGTAGGGAGATCTTCAGGAAATTCATCGTGGACTGGATCATCAGAGGTACAGGATGTTGTGGCTGTCTGTAAGTGGGCCTCCCAGTACATTTCTGCAGGA

AAAATACTCTTGGTCGGATCTTCCGCAGGTGCACCAATCGCAGGTTCTGCTGTTGACCAAGTTGAGCAAGTTGTAGGGTATGTAAGCTTAGGTTACCCCTTCGGCATGGTTGCGTCGATTTTGTTTGGAAGGCACCATAAAGCTATTTTGCA
GTCACCTAAACCAAAGCTCTTTGTAATGGGCACAAATGATGGATTTACGAGTGTAAAACAACTAAAAGATAAGTTGAAGATGGCAGCTGGACGTAATGAAATTCATCTTATACCTAGGGCTGGACACTTTCAGATGGAAGGCCCTGCCTAT
GATGAGAAAATGGCAGATTTGATTGATAAGTTTTCTGCAACCTTGTAGGTGAAGGGGCTTTGATCAAGAACATCACGTGTATATGCATTCCTTTCAAGTTCAAATTTCAGAGTTTAGTTTTGTGCCTGTCTTAATGTCAATGCTCAGCATGCT
GACATTGCGTCAAATTTTAGAAGTTGTTTGTACAATTACTCTTATTGTTAAGATGGCAATTATGAATTTAATGTTACCGCACTTACTCCTGACGACATTGAAATTGTGTCACATCATATGGCCACTGTTACTAAATCAAATGGAAGATCCAGC
AATACAAATTAAATGCACTTATAAAATAGTCATTTGAAGTATCGTGATAATAAACACCATGTCTTTATAATATTCATAAAGAAATGTCACATTTTGTAGAAAATTACAAATTGCTGAACATAATATAGATTCGTTTTTAGGTACTTTTGAGA
AACCTAAAT

c37446_gl

AAAGAATGCGTTATTCTTCTTCTAATTATTATATTTATAATATTCTACAAATCTGACGACAAGATGACCTCCTTAACCAGAACTATCATCGTTAACAGTCCATATTAGTGTACACAGAAAAAAATTCAGACTAAAGTTTTACCTAACCATTCA
TTTGTTCGGGGAGCTCCTGAAACAAAAAAATGGAGCTGCGGGGAAAAAAAATTGTGCGGATGCGAAGGCTATCCATCAGAGAGAGTGTGTGTCTTTACCCCAGTTCTCCACCACGGCCTTTTCCCCCTTTACTACTTGATAGACTAGGAAT
TAGGTTCTACTTCAATCTCCGAATCCTCCTGGGAATCGAACCCTTCACCCAACAACTCTTCCCAGAATACATCCGCGGTTTTAAGGTCCAAACCCCCGTTGGCAGCAGGGTTCTCTGCCACCGGCATGGAGGGCTCGCCCGTGCTTATGTAT
AGGGGAACTGAACTGTCGAAGGCCGGCTCGGGTTCGAACCCCTGCAAGTGCTCAAATTCGTCCGGGGATAATAACCCTTTCTCATCTCTAGACTGAAAAGCCTCTTCCGGTTGAGGATCCACCTGTCCCAGAGTGGACAAGAATGCCTCCA
AAGGCGTCGTGGTGGAGGGCTCCGACGCAGTTGAGGTGGAATTATTGGAATGGTACTTCACAATTTGGACCTGCGAAGAAGATGACTGGGATTGCAACTGGCATTCCGGCGCTTCGCCTGGCTCTCCCTCCCCCTGGGGCAGCCTCCGCCT
CTTCTTGGCACTGTCGAGTTCTTTAGCGACCTGGTTTTTTTGTGCCAGCTGGGAAAGAAACGTGGGATTCCGCATTGCCCTGGCAAGAAACGTCATCATCTGCTGCTGTCGGTGCTCGTTGGCCTGAATGCGCTGCCCCATTGCTTGCATCTG
GTTTCTGGTGGACTGCTGCTGGTGTCTCAGCCTCACCACTTCCATCATTAAAACACTCTTATCCCTTTTCAGACGCTCTATTTCTCCCTCCATTCCGAACTGCCCCACTTCAACGAAAGCCCCAAGGCCTTGCTGCTGCTGCTGCCCTTGCCCC
TGGGACGGTGGCCGCCGCCTCTGAATATGCTTCAGCAAGTGCTTCTGCCCTCTCAGAAACCCTTCGTTCGCAAATTCCCACCTGTCCGGATCCACCTTCCGAAACCCATACGTATTGAGTTGCCGAACAAAGCTCGAGAAATTGTTGTGCTT
GAAGTACTTAGGAAGCAAATCGATGGAAAATTGGTGCGAATTCCAGACTATGAAGCTATTGTTACCAACGCTCCACGAGACCACTGAATTGGTCTGAGGATCTTCCACCATTTCGTAGGTTTTAGTTAGAAATGGCGGTGGCCTTGTGCTCT
GCAACCCCTCGATGGGCTGAGGGGGGCTCCTCGACTCCATCGCTTGCAGTTCCTCTTTGATGATGGAATCGCCTTCGCTTCCTTCACAATAGTTCGAATCCATGACTGATGCCCCTGCCGTTGCGGTGCCCCTGCAAATCAAGTAAAATTCA
CATGAGCACACATCAATTCATAGCGCCTGAATTCGGATACCAATACAGATTTTTCAGCAATACAGAAATCCTTGAAGGTGTTGGTTGCAAGCATCATGTTGAGCCAGATACTGAAATAATTCAAAGCAATCTCCAATTTCCTGAACCAGTCT
TTGTTAGATAATGTAATTGCCGAGCTCTCCCGTAAGCGGGAAGAAGAGTACTACATTCAAACTTGCGACAATCGGAATGATTCCATATATATAGGGTACCACAGAAGCTTCTAGGCCTTTCGATTTTTTTCTTTTCTTTTTTCGTTCCCTTATT
TGCTTCCCTTGTTGCGATCTCCTCGGCCGGGGTTTGGGAAAAATG

c13349_g1

AAAAAAATTCTTCTTGAAGCCAATTTATGAAAGTATCTGGCTTTTGAGCTCTTTGAAAATTTGATGCTTGAGGTTTCATTCTTGTTCCATGCCTCGACATGGCCTCTACATGTCTTTCCATAGGGTCAAGCACCTCAATGTGAGTAGTGATAT
TTACTATTCCATCTTCAGAAGGATCAAGTCTTAATGTCCTTTCTAAGTTTTTGATTCTCCTATTATGGACTTAATCTAGATTTACCCTACCAGCTATGGGCATCGTCTTCCATTTCATCACCCACTCCCCCCCTTCCCCTCTCCCGCCAAAAGCA
AGTCTTCCTTAATATAGTAACCAGCTGTCCCATCCTGCAGTCAAACTGAGATTTCATGCAATCAAATCTAAACAAACTGATCCTGAAAAGAAGTGGCAATGGGAAATGTGTGTAACAAAAAAAAGAAATCTGAAACATAAACTCAAATGG
GCAGTGGCAAACAAACACCAAAGGAAAAACAAAACTTCATTAAATGACCAGTCTGCTGAAATTCATACGAACTCCCTTCTCCAAGACAAAACTCCTTCAAGGAAACCCTATTTTGGCTCTCTTCTCCTTCGTACTCCGCTATGCTGAAACTT
TTCCTATTCTCTATTCCCAACAGTCTTTTGCTTTTAATTCCTATTCGCTTGCCCTTACATTTTTAAGGAAAGGAAATACAAGGATATATAACATTAGCAAGTCAAAGCAAAAGTGGAAGGGTAATTATTGGTAATGTAGGGTGTTACATAGG
GCGGGGCAGTGTTTAGATCAGTTCCCTGGGCAAATATACAGCCTTGTCTCTTTACAGTATTCACCATTTCAATTGCCATGCTATTAGCATTTGAGTGTAATTGACTTGAGGATGTTTCTATTCAGATGGAACCAGGCCTTTCAATATCTTCAT
CAAGAAAGTGTTTTTTGTTGAATTCTGATGTGAAATTGTTGCAATTGATTGAGTGCAGGATGGTTGTATTGTGTGCCCA

c32969_g1

TTCTTAACTAAAAATTAATGATGTGAAAGGACATATTACTTAGATATATAATAATTCTATTCAAATCTTTTTAAATACATATTTTACTATTCTAAATTATACATACATCGAGGCAAGCAGGGAGGGTAGACCGTCAAATTTAGTCGTTTGTTA
CGGTGATCGTAAAAAATTATCGACTTTTTGGTTGACGCTATTAGACAGAAATTTCATGCAGATGGGAGGCGCTACTTTTGTGAGGGCTAAAATAGCAGACGGAACAATCCAAATTCCTTCGCCACAGATGAAACCAGACGCCATTACCGGA
ACGAAAATATCTGCTCTCTTGGAATTGATTTTGTGCCATGCGAACACAATGGAGGTTCCCAAACACATATCAATGGCGAAGTAGGGCCCCACGTAGAAGGGAATCGCCATGGCCATGGGCAGAGGAATATAGCAAGACACCCTCTCTGGC
AATACATCTCGCAAAGCATTGATGACAAGTGCAAAAGCAAAGGCGCCGAAACAAATATCTAAGCAATGGCTCGGGAGAGCCGAGAATCCTTCCACGCCTAAAATGGCCATGTTTCTGTAAATAAGAGCAAAAGGGGCTTTGTATTGCCCC
TGTGGATTCCCCACGTCGAAGGACTTGTAAAAGAGCCAGAAAGTGAGCGGCGCAATTATACATCCCATCGCATTGCCAACAAGTTGGCTCACAAACATCGATCTCGGAGATGAAAGCGTCAGGTGCCCGGTTTTGAAATCGTGCATTAAAT
CCGCTGCTGTATGGACAAGGGACTTCACTGTTCCGCACAGAGCTAGGCTAACAAGAACTCCCCCATGGTCTTCCCCTGCCCATGCTGCGTATACAAATAACGCAACCTTCCCATAATTGTATGCCAAGTTCTGATCTGTGAGGCCGGCTCCA
TATGCATTACAAAACGCCAGCACAGGAGCAAACATATAAGACACGAATACGTAGTACCACTTCATGTGATGGAACATCTGTGGGAGGACCCCTACGGATACAGCAGCCAGCGCTACATAACCAGATGCGGCTATCCAAACTGGTATGGTG
TCATTCACGAATATTTCGTTTTGCCTTTGCTCGTCTTTGGTGGTTGCACCGCCATTAACCTCATGCATGCCATTCAGCGTGTTCATTCTTTTCCTCCACTCCCTGTAGACGTTGCTCCCGGTGATCCACAATATTTTCACAAAATTGTAAAGCCC
GTCTCCTAAAATAATAGCAACACACACAAAAACCTTGAATCCATTAAGGCCCTTGATACTGCTTTCGGGAAGATTGTTGGGGTACCAGTCCCCTTCGCGTTCGCTAATGAGAGGCCACATGATACCCCATGAAAGGATCCCTCCTAAGAGC
ATAGAGAGATTGACCAGATGAGGGCAAATCATCCCTGCTCCCACGTATGTAAGGCTGAAGTCGGAGTAAAATCTCTGTTTCCAAGCTCTCAATCCAAAAATTGGAAAATTTACAAAGCCGCAATCTTCTCCGGCGGTGAAAAACCATTTGA
AAAAACCCCAAAGAAAACTCAACGTGAAATATTTTAAAAAGCATCGAACTTGCTTCCTTGCCAGTTTATCTTTGTGAGGTGTGTGGAAGCCGTTGATGAGCACAGCTGTTGCTGTGCCGCTCGGATAAGTAAGCTTGTAATCAACGATCAG
CACCTTTCGAAGAGGCACCAACACCAAAATGCCAACAAAAGTAACCAAAAAGAGAAATCCACTTATCCACCCAACTCCGGGCTCCTTTACTGTGTCTGGGGTATTACCGACGCTGTTAATTTCAGCCAGTGTATAGATTTTACTACTCATTC
CCAACAAATAGGAGCCAAATCCACCACCATAGGCGATTGAATAGCAAGCCACTACACATGTTTGAATAACTGTATTCTCTTGCTTGGTGAAAGGAGTTTGTAAGAAGCCAATCTTCTCCAAAATTTTGCTCCAACTTTTCATCAACACGAAT
CCTAAGAGCCCTGCAGAAACATTCATCGCGGGAGCTAGTCCAGTAGTGAGATTCAGATTCATGACTATAATACTGAAGAGGGTGCCGATCAGAAAGCTCACAACCAACCCCCGGAAGGATAACTGCTCTTTCCATGAAGGCACAACTTCTT
CGATATTTACTTCCCTAATACTCTGCTCATCTTTTCCAACCCACTCTCCCTTGCTTTCTTTTCTATTAATAATCTCTGGGCAATCTATATTGCAATTCACCATAGGCTTTTCTACTTCTAATGATGTCTCATGAACAATCCTCTTGACAAGTCCA

c24639_g1	TGTGCATCATCCATGTCAAAGGCGCTTACACTTCAATACTACAACAAGGCTAATCTTCTTTATTCACTATGAATGAACAAACACTTAAATGGGAAAAGGCTTTTCTTGCCGGAAATTCAGCACCTTGGGAAAGCAAGGAACGACATCTGCA<br>TCAATTGTAAGAGCTGTTGAAAGCATCTTAATCCTAGAATCTTACCTATGAGGAAAGACATCTGCAACAAGTGTAAGAGCTGTTGAAAGCATCTTAATCCTAGAATCTTACCTATGAGGAAAGACATCTGCAACAAG<br>GCTATTTACATCATTCATAGTTTTAGTTTCAATTTGCATAGGAGAAGCAGGGCGCCTTTCGAGTTTCACTCCACCCGAACCACAACCAGAAGAGGAGCCGACTATTGATTTGCCCGGTGGAACGTGGGCAGCTTTGAAGGGGCTCGTCAAT<br>GCTCGAAAAGGCGAGAGCATGAGAGGCGTCACAGAATTGAAGCACTACTTGCACAGTTTTGGATATCTCCTCGCCCAAGTACCAGTGCGCAATTTCACAGACTGCTATGATGACAGAGTTGAGTCGGCGGTGAAGCTTTATCAGCACAGCT<br>TCGACCTTCCAGTCACAGGAAATATTGACGAAGAAACCCTCTCGCAGATCATGACGCCGAGGTGCGGCGTTGCTGATATAATCGATGGCACTTCAACCATGGTGGCTCCAAACCGAAGCCACTCCCGTCCGGCCTTAGATAGTACAGTAAA<br>ACACTATTCTTTCTTCAACGGTACCCCCAAATGGCCCCCGGGCAAGCGAAATCTTACTTATGCATTCTCTCCCCTAAACAAATCGCTCGGAATCGACCAAGCCCAGGTCCAGACTGCGTTTTCCAGGGCGTTCAAAAGATGGGCTGCAGTGA<br>TTCCCGTGACGTTCACAGCAACAAAAAATTACAGTGGCGCTGACATAAAAATTGGCTTCTTCAGTGGAAATCACGGGGACGGGCAACCGTTCGACGGGCCTTTGGGGACTTTGGGCCATGCTTTTTCTCCGCAGAATGGAAGACTGCATAT<br>GGACGCAGACGAGCGGTGGAGCTTCAATTTGGAGGAAGAGGATAAATCGGATGCTAAGGCAATCGACCTGGAGTCCATTGCCACGCACGAAATAGGGCATATTTTGGGGTTAGGGCATTCTTCCGTGAAAGGCACTGTAATGTTTCCCAC<br>CATCTCCCCGCGCACTAGAAAAGTGGACCTCACTTACGATGATGTGTATGGAGCGCAAGCTCTGTATGGACCCAATCCCAATTTTGATCCTTCTTTGGTTTCCTTTGATGCCGCCGACCAATTTGCTCAAAACACTGCTCCTCCTCTGCCTCT<br>GCCTCTGCTCCACACCATTTCATTTTATTTCTTTCTCACCTCAACCTCATTCTTATGTGTTTTTTCCTGCGGGGGATTTATCATCCTCCACTTACTGTAAACTATTTTTATTTTTATACCACTGTCACTGTAACTGTAAATTATTTATTTTTTTTTT<br>ATTTTTTTTTT
c31449_g1	CACAGGAAATATCCATCCATTTTCTTTCAAACAAATGCATTACATATATAACCCTGAAAGACTTGGGCACATGCCCAAGTGGGTAGGGTGAACCTTCTTTCCCAATACGCAGAGAGGACATTATTGGGATTTCACTTGTTTACGGGGCTTGT<br>GACTCCTCCTGCAATTAGGTGTCATAAGTAGGCTTCCAGGATCCCAGGGCATGGCCTCCTGCGATGGATGTATACTTCATACAGGAAAAAAATTCCCCAGCCAAGCTTTTAGTTCAACAATCCTGATAAAAATGGTTTACCTAAATTAAAG<br>GAACAAGGAATAGAAAAAACCAATTTTTTTTTAGTGGAAACAATATTCATAAGGCTATCAAATGATCCAATAGCCTCTCTTGCCAATAGCCTGGGCTGATAGGGTGGCTTCGCAGAGATTATTCATGATGCCCAGTTATTATTCTGAACCAA<br>CAGGGATGTGTATATCTCTGTATCTGCAAATGTATGGAGCATGATGACCTACATAGGGTCCTGCCCACCTGCCCTCATCAATGTAGCTTGCATTCCATACAGAGGCATACAAAAACATGGGTTTGGATGGAAAATTCTCTCCCTCCTTAGCC<br>TCTTCACTCCTCACAATATTCCCGGCGATCAACCATTCAGTCCTTTGAGGGCCCCATCTGATAGGATACTCATGGAAGTCCTCGAAGCAATCAAATCCAAGCTGATGTATCTGTTCTCTGTTCCCAGTTCCATCTGTGTAAAAGTTAGTCTGT<br>ACAATGCCCTTATCCTTGCCTAAAAACTCAAAGTCTATCTCGTCTTGGGTTTTGTCCCCTTCAAGGGATGAAAGATAGAAGCTGCAGTTGATTCCACTGGTTTCTCCATGTGGGCACTTGATTCGAGAACTGAAACTGCCAGATCTGAATCTC<br>ATTAATGTTCTCCACCTTCCTCCTCCTCTCTCATCAAATGTAATGGTAATTTCTGCTTCCTCAGGCTTGTGATGACATACCTCAGGGGTGTAATCCACTGCAATCTCTCTAAGCCTCTTTGTTTCAAAAATCAACGGAGGAGCTGAATTTTTTT<br>GTAAGGCAAAATCTTGAGATGGGCAGGTCATCAGATGGGATTTCTCTCCAACAACTTCTTCCATGCATTGGAATGATGTGTAGGATGATGACTCTGTAGGTGCAGCCATAGTTAATGGTAAAGGAATTGAAAGGAGAAACGACGACAATA<br>TCCAGCAATGAAGGGCACCCACAACCTGGATCCAACCCTGCCTGTAATGCGTATTATATAATAATGTCTTCGCTTTCACCATTTCTACCAATACCTGTAAATGCGAGGTC

Table S17 Function annotations for 14 transcriptome sequences against four protein databases (Nr, GO, KO and Swiss-Prot).

Gene ID	Nr	GO	KO	Swiss-Prot Description
c23789_gl	hypothetical protein	metabolic process	-	-
c28921_g1	-	-	-	-
c61083_g1	-	-	-	-
c32904_g1	putative plastidial w-3 fatty acid desaturase	lipid metabolic process//oxidation-reduction process	omega-3 fatty acid desaturase (delta-15 desaturase)	Omega-3 fatty acid desaturas, chloroplastic OS
c33855_g2	hypothetical protein	motor activity//ATP binding//protein binding	myosin V	Myosin-11 OS
c61438_g1	-	-	-	-
c32552_g2	predicted protein	cellular protein modification process		
c23898_gl	-	-	-	-
c30526_gl	-	proteolysis//fatty acid metabolic process//metabolic process	-	-
c37446_gl	-	regulation of transcription, DNA-templated//cell cycle	heat shock transcription factor, other eukaryote	Heat stress transcription factor A-2e OS
c13349_g1	-	drug binding	-	-
c32969_g1	-	transmembrane transport	-	Metal-nicotianamine transporter YSL3 OS
c24639_g1	matrix metalloproteinase	proteolysis	-	Metalloendoproteinase 1 OS
c31449_g1	probable xyloglucan endotransglucosylase / hydrolase protein 30-like	carbohydrate metabolic process	-	Putative xyloglucan endotransglucosylase/hydrolase protein 1 OS

Table S18 Primer sequences, annealing temperatures, PCR products sizes, and gene ID of 14 nuclear loci used in this study.

Locus	Gene ID	Forward (5'-3')	Reverse (5'-3')	PCR product (bp)	Annealing temperature (ºC)
T8	c23789_gl	AGTGCCGCTGTTCGTTTC	GCCGCAAGATTCCTATTC	370	59
T26	c28921_gl	CGGAGCCTCACGAGATTA	TGATACACCCTGAAACCA	329	57
T82	c61083_g1	CTTCAGTAGGGATGGTTC	TCACAATCGTTGGAGGTT	329	57
T140	c32904_g1	TCTACTGCTTCTTCGTCCTT	TCGGCTCAAACTTGTCCT	374	57
T147	c33855_g2	TGCCCTACAGGAGATGGA	CAGTGTCTGCTGGCGAAT	391	58
T161	c61438_g1	TTTCCCTGTAAAGCACCT	TGTTGGCATTGACCTCCT	428	55
T173	c32552_g2	TCCCACAAATCTCACAAC	CTTTCCAGCACATTATCC	459	57
T203	c23898_gl	ACCCACAACTACAGACGC	ATGCCACCCTTTACAGAT	449	56
T212	c30526_gl	GCAAGTTGTAGGGTATGT	TCAGGAGTAAGTGCGGTA	483	59
T222	c37446_gl	ACCCTTCACCCAACAACT	AGGTCGCTAAAGAACTCG	447	59
T235	c13349_g1	GGGTCAAGCACCTCAATG	GGAGAAGGGAGTTCGTAT	418	58
T249	c32969_g1	AGAGCCAGAAAGTGAGCG	AACCACCAAAGACGAGCA	467	58
T275	c24639_g1	AGGAGCCGACTATTGATT	ATTCCGAGCGATTTGTTT	454	58
T293	c31449_g1	AGGGATGAAAGATAGAAGC	AATGGTGAAAGCGAAGAC	477	58

Table S19 The prior distribution of parameters for all scenarios in the simulations of DIYABC and fastsimcoal2.

Parameter	Distribution	Minimum	Maximum
Effective population size			
N_F	Uniform	200000	800000
N_Y	Uniform	10	40000
N_1	Uniform	10	30000
N_2	Uniform	10	100000
N_A	Uniform	10	100000
Migration rate			
m	Log uniform	1×10^{-8}	1×10^{-1}
Time of events (in generations backward in time)			
t_0	Uniform	10	40000
t_1	Uniform	10	60000
t_2	Uniform	10	100000
t_3	Uniform	10	200000
Mean mutation rate			
μ	Uniform	1×10^{-9}	1×10^{-7}

N_F and N_Y, current population sizes of *T. fargesii* var. *fargesii* and *T. fargesii* var. *yunnanensis*; N_A, ancestral population size; N_1 and N_2, population sizes between ancestral population and current population of *T. fargesii* var. *yunnanensis* and *T. fargesii* var. *fargesii*, $N_F > N_2$ and $N_Y > N_1$. *m*, population migration rate between *T. fargesii* var. *yunnanensis* and *T. fargesii* var. *fargesii*. t_0, t_1, t_2 and t_3, times of population changes, and $t_3 > t_2 > t_1$, $t_3 > t_0$.

Table S20 The geographical records of *T. fargesii* var. *fargesii* and *T. fargesii* var. *yunnanensis* used in the ecological niche modeling.

Taxon	Records	Longitude (°E)	Latitude (°N)
var. *fargesii*	1	109.61	28.56
	2	107.15	28.87
	3	107.14	28.95
	4	110.47	29.05
	5	107.10	29.20
	6	103.40	29.50
	7	103.37	29.55
	8	114.50	29.60
	9	110.78	30.10
	10	110.90	30.20
	11	102.80	30.40
	12	102.58	30.62
	13	110.75	30.66
	14	110.30	31.00
	15	111.30	31.00
	16	116.04	31.00
	17	116.20	31.09
	18	115.77	31.17
	19	110.90	31.20
	20	110.09	31.29
	21	110.30	31.30
	22	110.80	31.30
	23	115.32	31.40
	24	116.30	31.40
	25	110.38	31.47
	26	110.50	31.50
	27	110.89	31.59
	28	108.91	31.69
	29	111.20	31.70
	30	109.57	31.75
	31	110.68	31.76
	32	109.51	31.77
	33	110.78	31.79
	34	108.60	31.80
	35	108.40	31.96
	36	108.90	32.07
	37	108.00	32.10
	38	109.80	32.10

	39	110.70	32.10
	40	109.20	32.12
	41	113.10	32.20
	42	108.09	32.23
	43	105.80	32.40
	44	106.80	32.40
	45	109.40	32.40
	46	110.70	32.60
	47	107.65	32.62
	48	110.10	33.00
	49	109.43	33.02
	50	105.28	33.03
	51	106.70	33.20
	52	106.33	33.26
	53	109.07	33.31
	54	106.10	33.80
var. *yunnanensis*	55	99.65	26.28
	56	99.30	26.86
	57	100.20	26.90
	58	99.41	26.93
	59	99.39	27.03
	60	99.15	27.08
	61	99.09	27.09
	62	99.26	27.16
	63	99.30	27.20
	64	99.52	27.25
	65	99.26	27.31
	66	99.01	27.51
	67	98.70	27.70
	68	99.70	27.80
	69	99.49	27.80
	70	98.80	27.90
	71	98.60	28.00
	72	98.70	28.00
	73	98.90	28.00
	74	98.59	28.09
	75	98.60	28.10

Table S21 Pairwise Pearson correlation coefficients (*r*) of 20 environmental variables.

Pearson Correlation	bio1	bio2	bio3	bio4	bio5	bio6	bio7	bio8	bio9	bio10	bio11	bio12	bio13	bio14	bio15	bio16	bio17	bio18	bio19	altitude
bio1	1																			
bio2	-.107	1																		
bio3	-.271*	**.898****	1																	
bio4	.366**	-.688**	**-.931****	1																
bio5	**.832****	-.456**	**-.713****	**.815****	1															
bio6	**.864****	-.065	-.017	-.030	.512**	1														
bio7	.402**	-.487**	**-.818****	**.967****	**.827****	-.059	1													
bio8	**.842****	-.389**	-.636**	**.751****	**.959****	.533**	**.766****	1												
bio9	.638**	.466**	.501**	-.448**	.122	**.809****	-.388**	.185	1											
bio10	**.852****	-.457**	-.699**	**.798****	**.996****	.544**	**.802****	**.967****	.161	1										
bio11	**.703****	.422**	.446**	-.404**	.195	**.872****	-.344**	.254*	**.971****	.229*	1									
bio12	.102	-.458**	-.249*	.105	.067	.213	-.061	.095	.115	.122	.027	1								
bio13	.012	.084	.354**	-.484**	-.321**	.306**	-.574**	-.247*	.470**	-.267*	.385**	**.704****	1							
bio14	.185	-.553**	-.533**	.507**	.362**	.053	.386**	.335**	-.122	.396**	-.200	**.757****	.202	1						
bio15	-.205	.664**	**.775****	**-.793****	-.565**	.072	**-.704****	-.524**	.384**	-.575**	.397**	-.427**	.294*	**-.813****	1					
bio16	.022	.027	.306**	-.448**	-.294*	.318**	-.550**	-.229*	.456**	-.240*	.367**	**.760****	**.974****	.224	.250*	1				
bio17	.172	-.466**	-.451**	.450**	.316**	.037	.343**	.308**	-.067	.355**	-.169	**.772****	.263*	**.986****	**-.759****	.283*	1			
bio18	-.024	.133	.410**	-.533**	-.377**	.275*	-.618**	-.271*	.482**	-.319**	.386**	**.710****	**.965****	.219	.281*	**.966****	.285*	1		

bio19	.185	-.425**	-.406**	.407**	.298**	.062	.305**	.291*	-.011	.339**	-.123	**.789****	.304**	**.977****	**-.733****	.325**	**.996****	.328**	1	
altitude	-.699**	.590**	**.825****	**-.905****	**-.961****	-.358**	**-.884****	**-.923****	.061	**-.961****	.003	-.165	.318**	-.493**	.695**	.290*	-.445**	.373**	-.416**	1

The coefficients (*r*) larger than 0.70 shown in bold. * and **, significant level at $P < 0.05$ and $P < 0.01$, respectively.

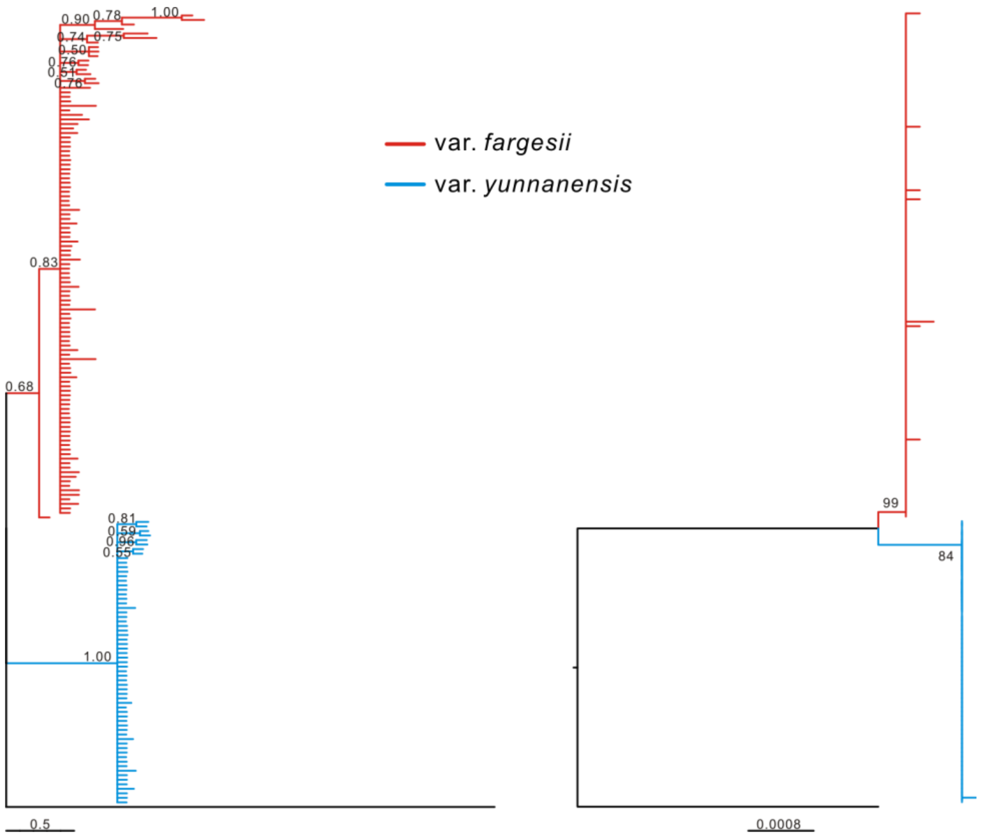

Figure S1 Bayesian tree (left) and Neighbor-joining (right) tree were constructed by the concatenated nuclear dataset using MrBayes and MEGA, respectively. Support values were showed above the branches.

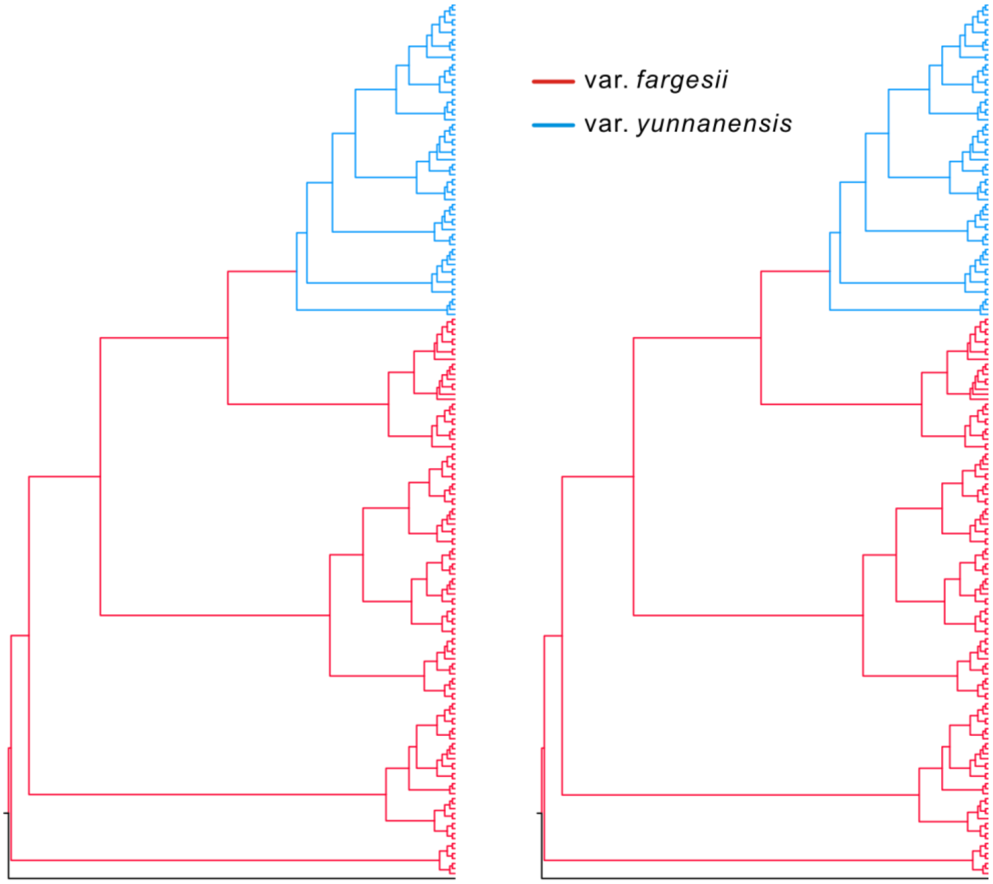

Figure S2 Phylogenetic trees were inferred using BEAST based on the partitioned nuclear datasets, the first dataset (14 nuclear loci) (left) and the second dataset (12 nuclear loci) (right). Posterior values of all clades are lower than 0.5.

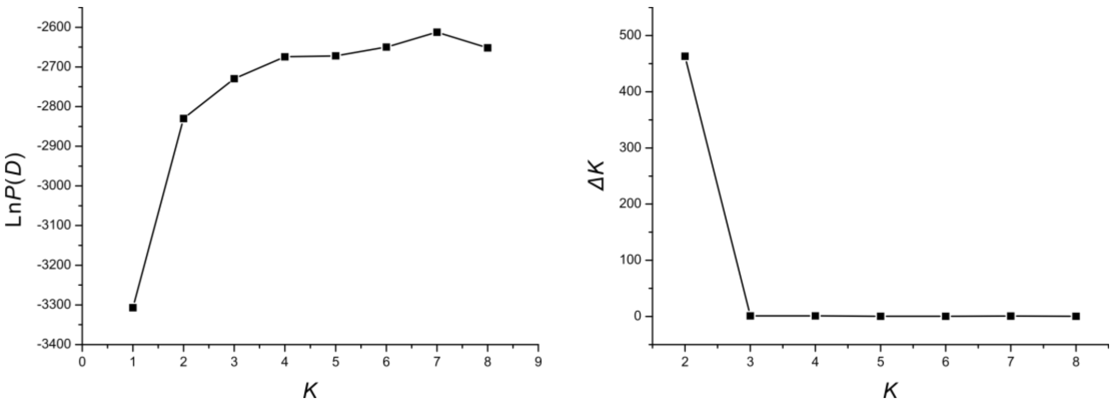

Figure S3 The most likely number of clusters (K) inferred with LnP(D) (left) and ΔK (right) statistics implemented in STRUCTURE.

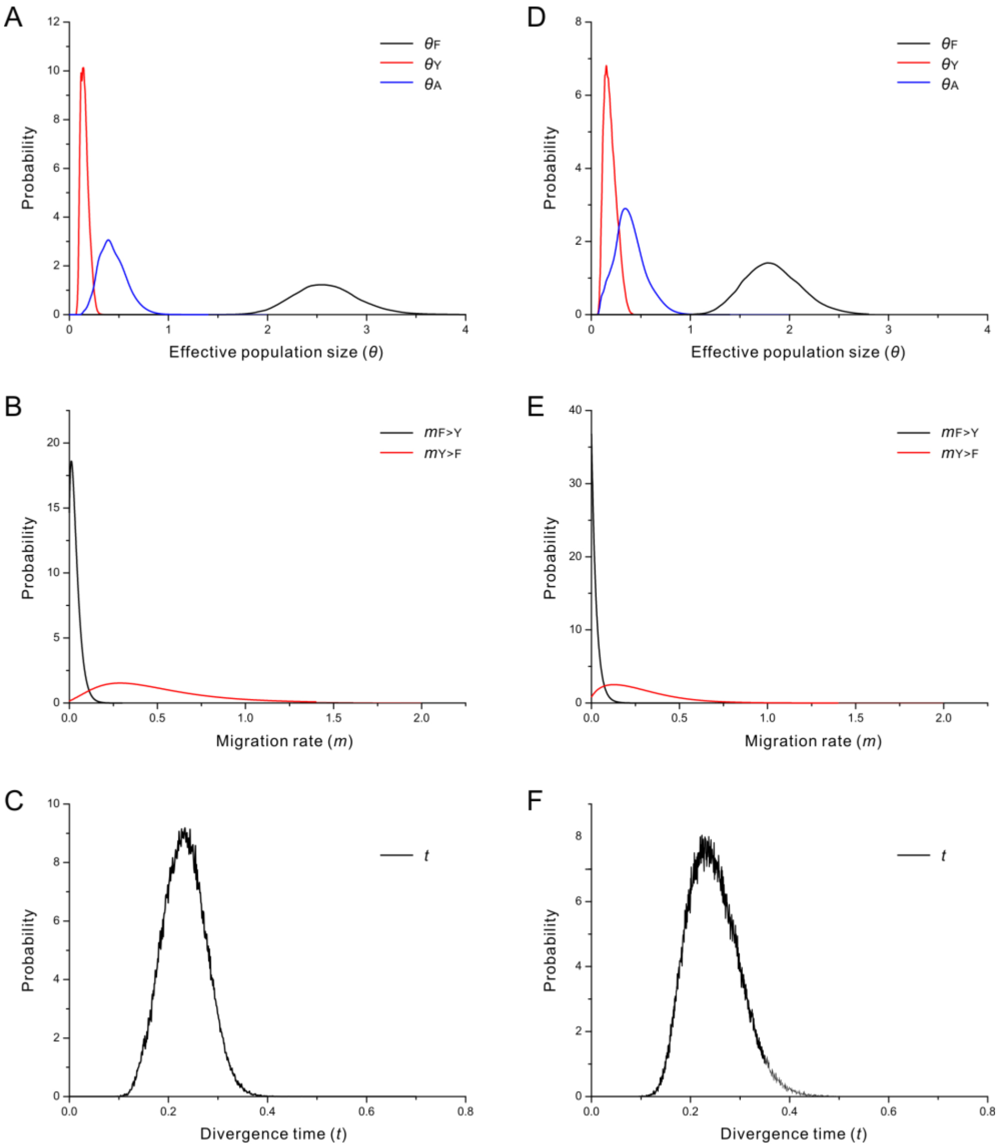

Figure S4 Posterior probability distributions of effective population size (θ), migration rate (m) and divergence time (t) between two varieties estimated separately using IM model based on the first dataset (14 nuclear loci) (left) and the second dataset (12 nuclear loci) (right).

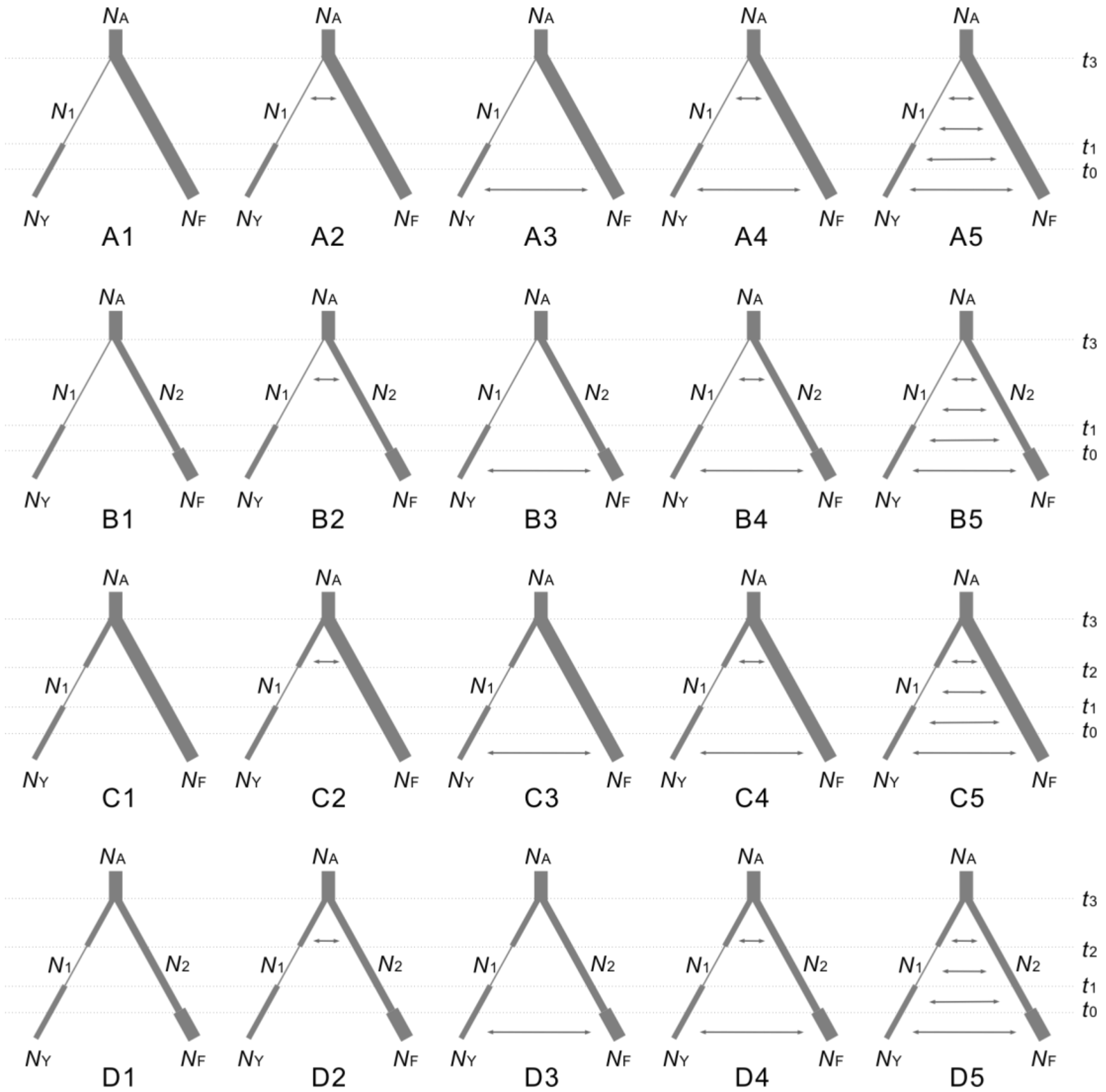

Figure S5 Four basic scenarios (A1, B1, C1 and D1) with different migration models for the divergence and demographic history of *T. fargesii* var. *fargesii* and *T. fargesii* var. *yunnanensis*. N_F and N_Y represent the current population sizes of *T. fargesii* var. *fargesii* and *T. fargesii* var. *yunnanensis*, and N_1 and N_2 represent the population sizes between ancestral population and current population of *T. fargesii* var. *yunnanensis* and *T. fargesii* var. *fargesii*, respectively. N_A represents the ancestral population size. t_0, t_1, and t_2 represent the time of population changes and t_3 the divergent time. A2–A5, B2–B5, C2–C5, and D2–D5 are the derivatives of A1, B1, C1, and D1 by adding migration parameters at different times, respectively.

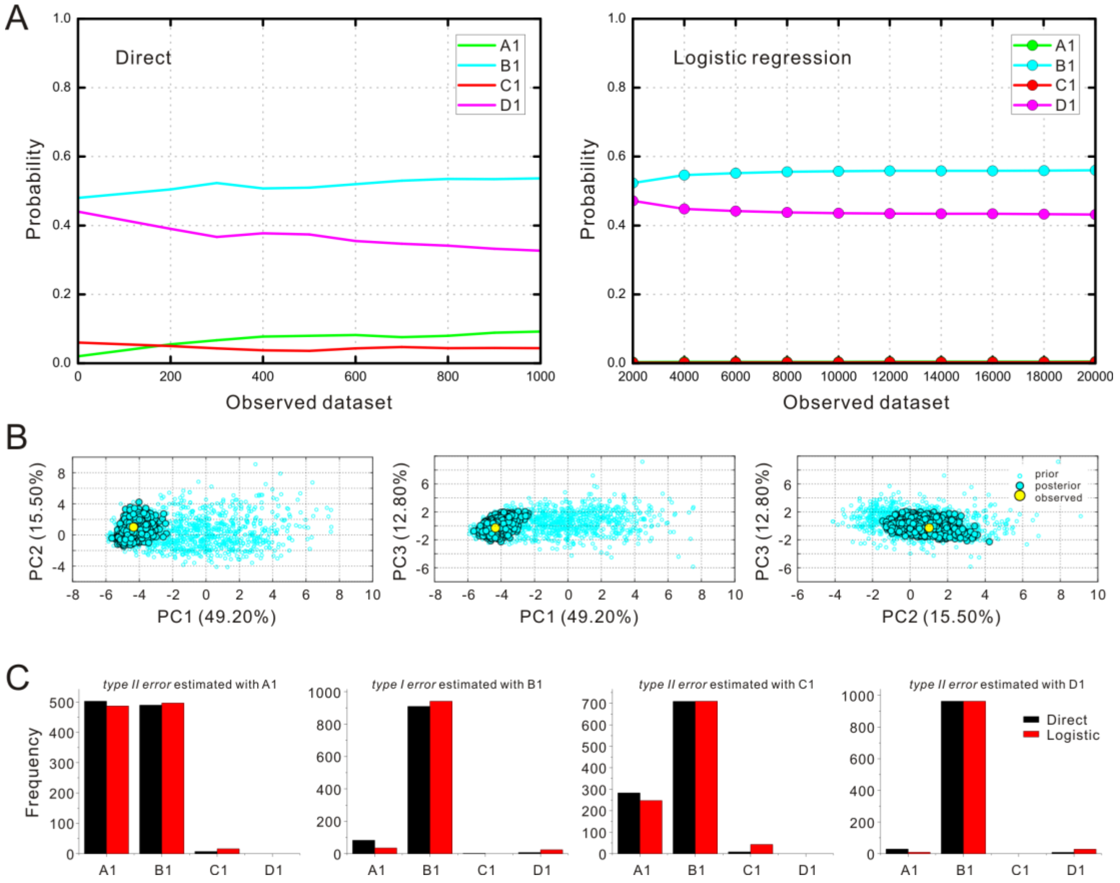

Figure S6 Posterior probabilities for four basic scenarios (A1, B1, C1 and D1 in Figure S5) (A), model checking for the optimum scenario B1 (B), and level of confidence in scenario choice (including *type I error* and *type II error*) (C) estimated using direct approach and logistic regression in DIYABC based on the first dataset (14 nuclear loci).

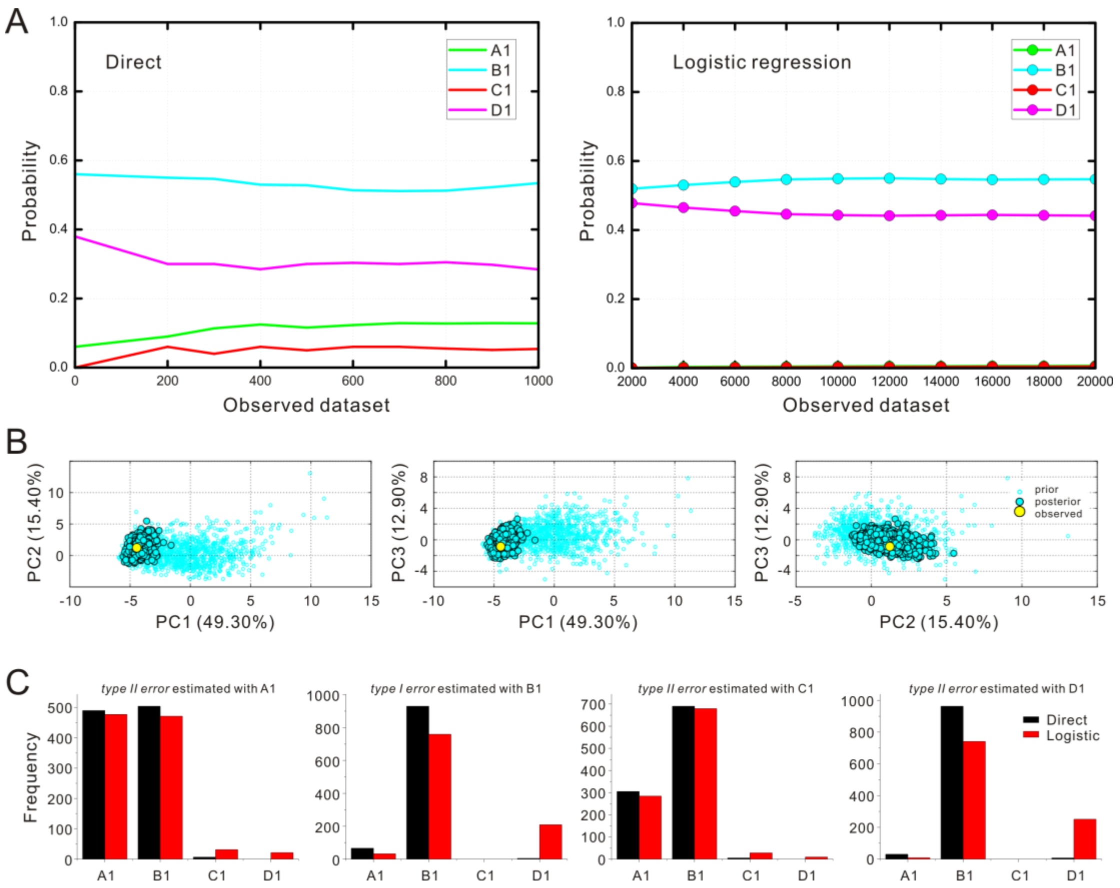

Figure S7 Posterior probabilities for four basic scenarios (A1, B1, C1 and D1 in Figure S5) (A), model checking for the optimum scenario B1 (B), and level of confidence in scenario choice (including *type I error* and *type II error*) (C) estimated using direct approach and logistic regression in DIYABC based on the second dataset (12 nuclear loci).

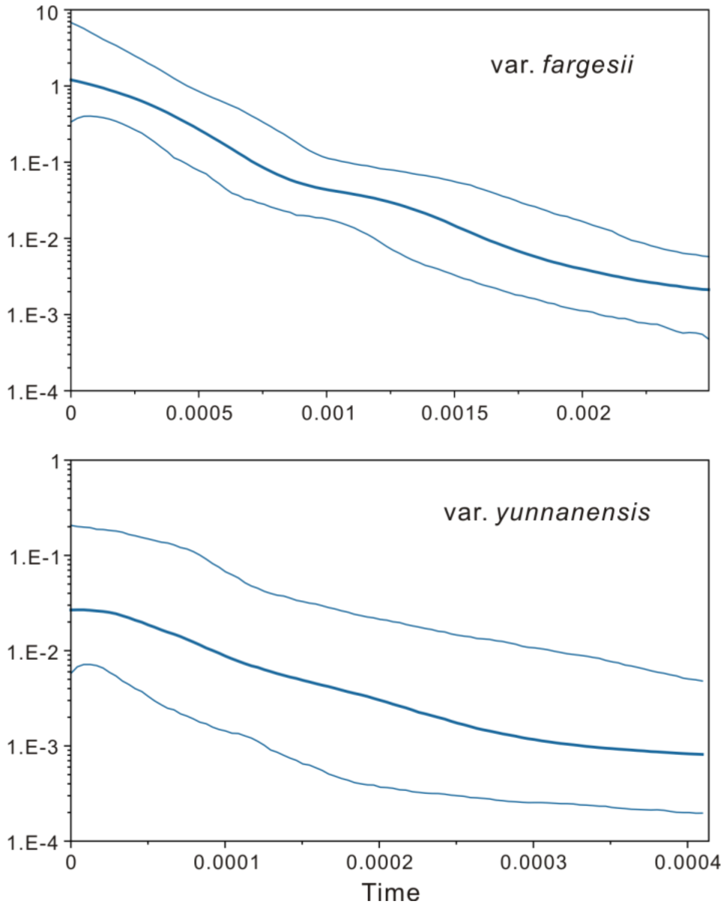

Figure S8 Bayesian skyline plot inferred for *T. fargesii* var. *fargesii* and *T. fargesii* var. *yunnanensis* in BEAST based on 14 nuclear loci. The bold and thin lines are the median posterior and 95% highest posterior densities of effective population size through time, respectively. The effective population size and time were not scaled using mutation rate.

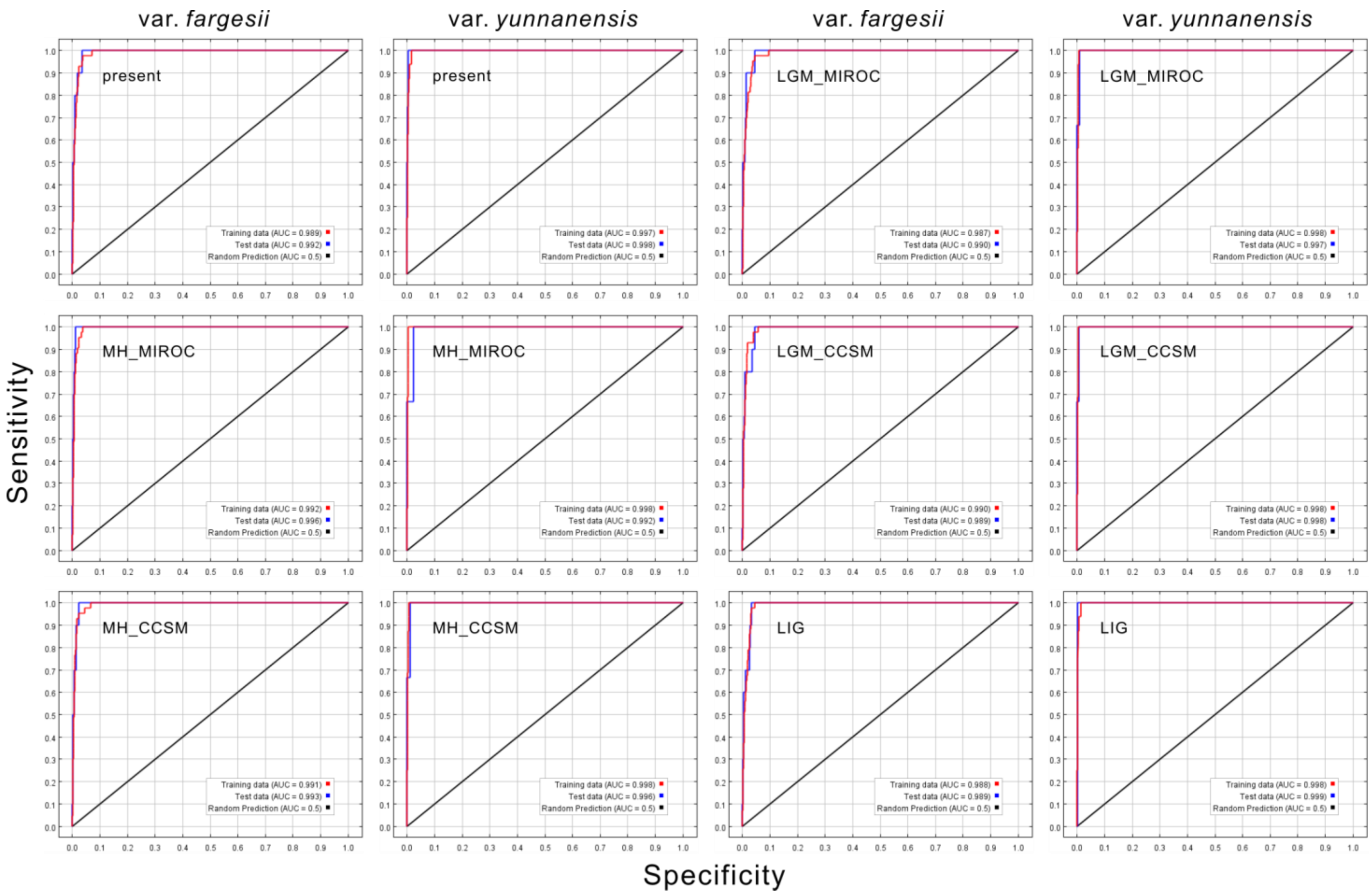

Figure S9 The ROC curve (AUC) for each predicted distribution, present-day, Mid-Holocene (MH, under MIROC and CCSM models), the Last Glacial Maximum (LGM, under MIROC and CCSM models), and the Last Interglacial (LIG) climatic periods.

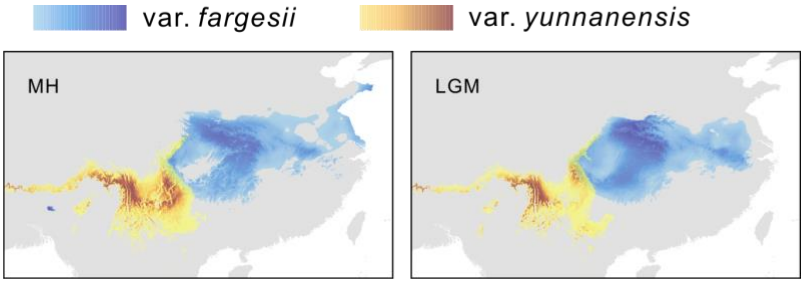

Figure S10 Climate niches during Mid-Holocene (MH) and the Last Glacial Maximum (LGM) under CCSM model were modeled and drawn using MAXENT 3.4.3 for *T. fargesii* var. *fargesii* and *T. fargesii* var. *yunnanensis*.
